# Exploring the feasibility of modeling next-day fatigue and sleepiness using digital sleep tracker data in neurodegenerative and immune-mediated inflammatory diseases

**DOI:** 10.3389/fdgth.2026.1752629

**Published:** 2026-06-17

**Authors:** Bing Zhai, Luan Chen, Xujun Ma, Clémence Pinaud, Meenakshi Chatterjee, Juha M. Kortelainen, Rana Zia Ur Rehman, Teemu Ahmaniemi, Stefan Avey, Yu Guan, Victoria Macrae, Chloe Hinchliffe, Silvia Del Din, Nikolay V. Manyakov, Robert Göder, Robbin Romijnders, Walter Maetzler, Ralf Reilmann, Svenja Aufenberg, Robin Schubert, C. Janneke van der Woude, Daqing Zhang, Wan-Fai Ng

**Affiliations:** 1 Translational and Clinical Research Institute, Faculty of Medical Sciences, Newcastle University, Newcastle upon Tyne, United Kingdom; 2 Computer and Information Sciences, Northumbria University, Newcastle upon Tyne, United Kingdom; 3 SAMOVAR, Télécom SudParis, Institut Polytechnique de Paris, Palaiseau, France; 4 ETIS UMR8051, CY Cergy Paris Université, ENSEA, CNRS, Cergy, France; 5 Let it Care, Paris, France; 6 Johnson & Johnson, Cambridge, MA, United States; 7 VTT Technical Research Centre of Finland Ltd., Espoo, Finland; 8 Johnson & Johnson, Buckinghamshire, United Kingdom; 9 Johnson & Johnson, Spring House, PA, Unites States; 10 Department of Computer Science, University of Warwick, Coventry, United Kingdom; 11 National Institute for Health and Care Research (NIHR), Newcastle Biomedical Research Centre (BRC), Newcastle University, Newcastle upon Tyne, United Kingdom; 12 Johnson & Johnson, Beerse, Belgium; 13 Department of Psychiatry and Psychotherapy, University Hospital Schleswig-Holstein, Kiel University, Kiel, Germany; 14 Department of Neurology, University Hospital Schleswig-Holstein, Kiel University, Kiel, Germany; 15 George Huntington Institute, University of Münster, Münster, Germany; 16 Erasmus MC, Rotterdam, Netherlands

**Keywords:** digital health, IDEA-FAST, immune-mediated inflammatory diseases (IMID), mental fatigue, neurodegenerative diseases (NDD), physical fatigue, sleepiness

## Abstract

**Background:**

Fatigue and sleep disturbances are highly prevalent in neurodegenerative diseases (NDDs) and immune-mediated inflammatory diseases (IMIDs). Conventional patient-reported outcomes (PROs) are subjective and prone to recall bias; Digital health technologies and wearable sleep trackers offer objective, continuous monitoring of sleep and physiology at home.

**Objective:**

This study evaluated the feasibility of using consumer- and research- grade sleep trackers to predict next-day physical and mental fatigue and daytime sleepiness in individuals with NDDs and IMIDs as an exploratory analysis, and examined whether machine-learning models could identify preliminary sleep features to inform future fatigue monitoring research in chronic disease populations.

**Methods:**

The IDEA-FAST feasibility study enrolled 134 participants (42 healthy adults, 39 NDD, 53 IMID) across four European centres. Over 3,062 nights, participants wore three sleep trackers (BedSensor, ZKONE, DREEM 2) and completed daily fatigue and sleepiness PROs at home. A polysomnography sub-study (n=28) validated tracker performance. Machine learning models using physiological and sleep-architecture features were evaluated with leave-one-subject-out cross-validation.

**Results:**

Sleep trackers showed moderate PSG agreement. Models demonstrated preliminary discriminative capacity for next-day physical fatigue in healthy adults (AUC = 0.75), driven mainly by respiratory rate and REM sleep duration. In NDD, physical fatigue AUC reached 0.62 under enriched training, with REM latency and deep sleep as key features. Mental fatigue prediction reached AUC = 0.66 in healthy adults; daytime sleepiness AUC = 0.66 in NDD. Findings should be interpreted as exploratory, as outcome binarisation using a global threshold may conflate between-person disease-group differences with within-person symptom variation.

**Conclusions:**

Wearable sleep trackers show feasibility for objective home-based sleep monitoring, with preliminary evidence supporting sleep physiology as a candidate predictor of next-day physical fatigue in healthy adults. Predictive performance in chronic disease cohorts remains limited, underscoring the need for larger, multimodal studies to establish disease-specific digital fatigue endpoints.

## 1 Introduction

Fatigue is a subjective experience of weakness, diminished energy, or persistent tiredness, recognized as a complex and multidimensional phenomenon affecting physiological, cognitive, motivational, and emotional domains, thereby limiting an individual’s capacity to perform daily activities [1–3]. It is commonly categorized into physical fatigue, characterized by reduced physical energy and impaired motor performance, and mental fatigue, marked by decreased cognitive efficiency [4–6].

In chronic diseases, fatigue frequently co-occurs with sleep disturbances, and together they substantially impair activities of daily living (ADL) and health-related quality of life (HRQoL) [7, 8]. These symptoms are particularly prevalent and disabling in neurodegenerative disorders (NDD) and immune-mediated inflammatory diseases (IMID), where they are strong predictors of reduced HRQoL and increased healthcare utilization [9]. Notably, in both NDD and IMID, fatigue and sleep disturbances often persist independently of primary disease activity, suggesting partially distinct and insufficiently understood mechanisms.

Neurodegenerative disorders (NDDs) are progressive diseases that damage the structure and function of the nervous system, leading to gradual neuronal degeneration and loss [10]. In conditions like Parkinson’s disease (PD), fatigue and sleep disturbances are especially prevalent and problematic. Studies have shown a strong correlation between increased daytime sleepiness and fatigue, with PD patients experiencing greater daytime somnolence compared to those without fatigue [11–15]. Additionally, fatigue is closely linked to sleep disturbances, as evidenced by assessments using the Parkinson’s Disease Sleep Scale. Patients with fatigue tend to exhibit more severe sleep-related issues [13, 14, 16].

Immune-mediated inflammatory diseases (IMIDs) are chronic conditions characterized by abnormal immune responses leading to inflammation in various parts of the body. These diseases can affect multiple organ systems, including the skin, joints, lungs, gastrointestinal tract, and nervous system. Fatigue is a pervasive and debilitating symptom in IMIDs. Though the relationship between fatigue and inflammation is not entirely understood, it likely arises from a combination of biological, physiological, psychosocial, and behavioral factors that vary over time and across individuals [3, 17, 18].

Despite its high prevalence and impact, fatigue remains difficult to measure and manage in routine clinical care because it fluctuates substantially over time and relies largely on subjective self-report [19]. Continuous and objective monitoring approaches could help capture these day-to-day variations and provide earlier signals of worsening symptoms. In particular, predicting next-day fatigue could enable proactive symptom management, allowing patients and clinicians to adjust daily activities, plan energy expenditure, or implement targeted behavioral or therapeutic interventions [14, 20–22]. Advances in wearable technologies that passively measure sleep and physiological signals offer a promising opportunity to develop scalable exploratory digital features for feasibility investigation for fatigue monitoring and prediction in real-world settings [23].

Neurodegenerative disorders and immune-mediated inflammatory diseases provide a particularly relevant context for investigating fatigue prediction. Although these disease groups arise from different biological mechanisms, both are characterized by high fatigue burden, frequent sleep disturbances, and substantial day-to-day symptom variability [9, 11, 12, 17]. In both conditions, fatigue often persists independently of primary disease activity and remains challenging to manage clinically. Sleep disturbances represent another highly prevalent and clinically relevant feature across both NDD and IMID. In HDs, alterations in sleep architecture and circadian rhythms have been linked to cognitive impairment, depression, and disease progression [7, 13, 24]. In IMID, sleep disruption is also common and may arise from both inflammation-driven circadian dysregulation and disease-related symptoms such as pain [25]. These shared characteristics make NDD and IMID suitable populations for exploring whether physiological signals captured during sleep can help anticipate next-day fatigue, while also enabling comparison of how the sleep–fatigue relationship may differ across disease contexts.

Standardised questionnaire-based patient-reported outcomes (PROs) are the most widely used tools for evaluating fatigue and sleep disturbance. While PROs provide essential insight into a patient’s lived experience, they also present well-recognised limitations for monitoring dynamic symptoms. Responses are inherently subjective and may be influenced by demographic, cultural, and individual factors; long-term completion is often inconsistent due to fluctuating health or disease burden; and recall-based reporting can lead to over- or underestimation of symptom severity, motivating the development of complementary objective measures [26].

Digital Health Technologies (DHT) have made significant strides in the past decade, offering health professionals the potential to capture objective, reliable, and sensitive-to-change measurements [27–30]. Wearable devices, such as fitness trackers, smartwatches, and health monitoring patches, are now commercially available and capable of providing real-time assessment of health indicators, offering personalized data on individuals’ health and daily activities. However, as these devices were primarily designed for use in healthy individuals, it remains unclear whether comparable performance can be achieved for clinically meaningful outcomes or whether such technologies are acceptable to patient populations. It is important to note that while the analysis of data from DHT has largely relied on basic correlation approaches, the potential to utilize this information for classifying patient health status has not been extensively explored yet.

Machine learning (ML) provides a natural framework for leveraging the richness of wearable time-series data by integrating multiple signals into unified predictive models [31, 32]. Such methods can identify patterns that are difficult to detect using conventional statistics and may enable the construction of digital biomarker instruments that are sensitive to clinically meaningful change [33–35]. Importantly, interpretable ML workflows can also provide insight into which sleep and physiological features contribute most strongly to fatigue-related outcomes, thereby improving mechanistic understanding and informing future endpoint development.

Expanding on these concepts, the IDEA-FAST project^1^ employs various home-based sensing modalities and technologies to pinpoint digital biomarkers associated with fatigue and sleep in 2,000 individuals with NDD and IMID [36]. The device measures include physical activity, physiology, electroencephalography (EEG), neurocognitive performance, and socialisation parameters. It is a five-and-a-half-year project that aims to select suitable digital endpoints and determine potential digital biomarkers using multiple sensing devices to assess fatigue, sleep, and activities of daily living for patients with Parkinson’s (PD), Huntington’s (HD), rheumatoid arthritis (RA), systemic lupus erythematosus (SLE), primary Sjogren’s syndrome (PSS) and inflammatory bowel disease (IBD) [2, 9]. Within this context, an important open question is whether wearable sleep measurements collected “in-the-wild” can be used not only to characterise sleep, but also to support next-day fatigue monitoring in patient populations.

In this study, we present a preliminary feasibility analysis examining whether sleep characteristics derived from home-based wearable sleep trackers can provide exploratory signals relating to next-day fatigue outcomes in individuals with NDDs and IMIDs, alongside healthy controls. We implement an end-to-end analytical pipeline that includes wearable data curation, extraction of sleep-related features, and participant-independent evaluation of predictive models. To support the credibility of wearable-derived sleep measures in these clinical populations, we assess agreement between wearable-estimated sleep staging and gold-standard polysomnography (PSG) annotations in an independent dataset. Finally, we explore the relationship between wearable-derived sleep features and next-day fatigue and sleepiness outcomes, using model interpretation to identify which aspects of sleep physiology and architecture are most informative across healthy and disease groups.

This work makes three key contributions. First, to the best of our knowledge, it provides the first comparison study of using wearable sleep technology to explore the feasibility of fatigue-related prediction in individuals with NDD, IMID and healthy individuals in home settings. Second, we introduce and evaluate a reproducible modelling framework for predicting next-day fatigue from nightly sleep tracker signals using participant-independent validation, reflecting realistic deployment scenarios. Third, by pairing feasibility modelling with PSG-based assessment and feature-level interpretation, we offer an integrated view of (i) whether the sleep signals captured by wearables are sufficiently credible in these populations and (ii) how sleep–fatigue relationships may differ across disease groups—insights that are essential for the design of future, adequately powered studies targeting clinically meaningful digital features.

## 2 Methods

**Figure 1:**
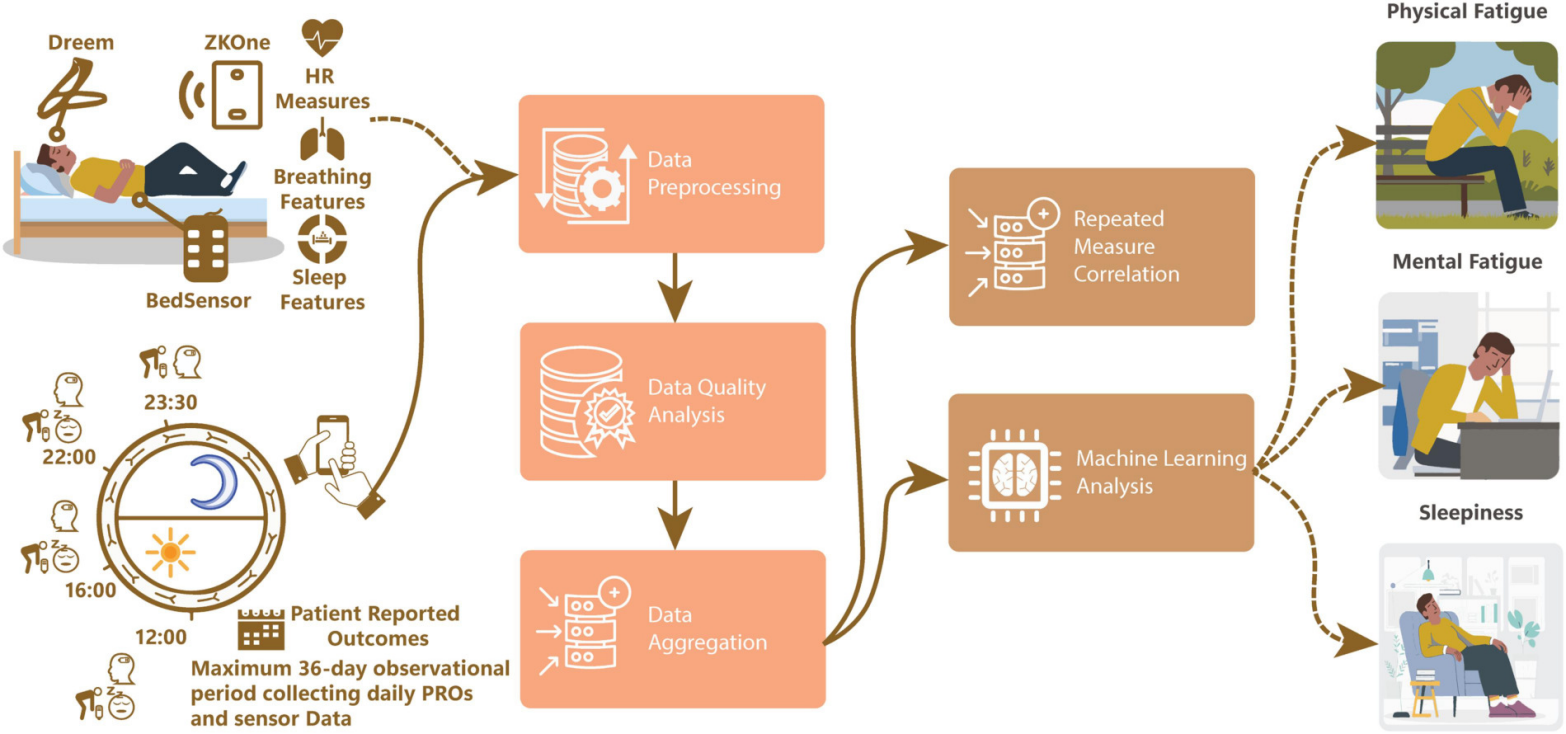
Prediction workflow for estimating physical fatigue, mental fatigue, and sleepiness in healthy adults, neurodegenerative disorder patients, and immune-mediated inflammatory disease patients in a long-term study with an up to 36-day analysis per participant. The dashed lines show that these data were used for training the model, while only sensor readings are used as input during prediction.

The overall study design can be seen in Figure 1, three main sleep devices (i.e., DREEM 2, ZKONE, and BedSensor) are deployed in a home environment to record nocturnal datasets separately. Additionally, a stress monitor application (SMA) is used to collect PROs from the participating subjects. The methodology comprised validation of commercial sleep trackers in patient cohorts through comparison with PSG, followed by data preprocessing and quality assessment. We then conducted PSG-oriented device validation and examined associations between wearable-derived measures and PROs, before applying machine learning–based modelling to enhance predictive performance.

As illustrated in Figure 1, we first assess the performance of commercial sleep trackers in patient cohorts by comparing their measurements with polysomnography (PSG) as the reference standard. We then describe the design of the feasibility study, including the study protocol, participant demographics, data collection and curation procedures, feature extraction methods, statistical analyses, and machine learning (ML) modelling pipeline. This framework is used to examine the relationship between nightly sleep characteristics obtained from home-based wearable devices and next-day fatigue outcomes across three cohorts.

### 2.1 Validation of sleep trackers

At the beginning of feasibility study, three commercially available sleep trackers were employed. Prior to conducting the statistical analyses and machine learning modeling, a device validation was performed by comparing the sleep metrics obtained from these trackers with those derived from PSG. A total of 28 participants undergoing PSG observation at the Schlaflabor, Department of Psychiatry and Psychotherapy, UKSH Kiel (as part of the IDEA-FAST feasibility study), were enrolled over a 2-month period. The cohort comprised 15 individuals with PD, 10 individuals with RA, and 3 individuals with HD (Table 1).

**Table 1:** Summary of data from PSG sub-study used for sleep tracker validation in IMID and NDD population.

Device	Polysomnography	ZKONE	BedSensor	DREEM 2
Age range	38–79	38–79	38–79	38–79
Number of participants (IMID/NDD)	28 (10/18)	20 (8/12)	23 (8/15)	24 (9/15)
Number of recorded nights (IMID/NDD)	56 (20/36)	36 (15/21)	44 (16/28)	47 (18/29)

PSG recordings were conducted using Somnomedics systems and included electroencephalography (EEG), electrooculography (EOG), and submental electromyography (EMG). EEG electrodes followed the 10–20 system, with C4, O2, and F4 referenced to M1, and C3-M2 as backup. PSGs were visually scored by an experienced technologist in the German Sleep Society (DGSM)-certified Sleep Lab of the University Hospital Schleswig-Holstein Campus Kiel using locally available analysis software and following the AASM guidelines. These scorings are regularly checked by another rater in the lab to ensure the highest scoring quality. Participants wore three consumer-grade sleep trackers during two consecutive PSG nights: (1) BedSensor,^2^ (2) ZKONE YOLI sleep monitor,^3^ and (3) DREEM 2 headband^4^ (detailed descriptions can be found in Multimedia Supplementary Appendix A).

To evaluate accuracy, tracker hypnograms were time-synchronized with PSG data, compensating for discrepancies (e.g., PSG clocks lagging internet time by up to 3 min). All data were resampled to 30-s epochs. Epoch-by-epoch comparisons between trackers and PSG were summarized in confusion matrices. Since ZKONE and BedSensor classify sleep as either “light sleep” or “deep sleep,” while PSG distinguishes between N1, N2, and N3 stages, N1 and N2 were combined as “light sleep,” and N3 was treated as “deep sleep” for comparison. When comparing DREEM 2 with PSG, two approaches were used: (1) analyzing N1, N2, and N3 stages separately, and (2) grouping them into “light sleep” and “deep sleep.” The latter method also enabled a unified comparison across all three sleep trackers.

The number of participants in the PSG study is shown in Table 1. Device/network failures and COVID-19-related recruitment challenges compromised some sleep recordings, reducing the dataset available for sleep tracker validation.

### 2.2 Feasibility study data collection: digital questionnaires and wearable data

#### 2.2.1 Feasibility study participants

The FS was conducted across six disease populations [2]. A total of 159 participants were recruited, including individuals with PD (n=25), HD (n=14), IBD (n=18), PSS (n=18), RA (n=24), and SLE (n=18), as well as healthy volunteers (HV, n=42). For analysis purposes, participants with PD and HD were grouped as NDD, while those with IBD, PSS, RA, and SLE were grouped as having IMID. Participants from these seven cohorts were recruited from four European centres: Universitätsklinikum Schleswig-Holstein Kiel (K, n=56), Newcastle University (N, n=57), Erasmus University Medical Centre Rotterdam (E, n=28), and the George-Huntington-Institute (G, n=18). Each participant was monitored over a four-week period, during which they used various sleep trackers, rotating them over four 5-day periods, in addition to other digital health technologies.

#### 2.2.2 Patient reported outcomes

During the study, participants were asked to report their mental and physical fatigue levels four times (at 9:00, 13:00, 17:00, and 21:00 local time) daily on a Likert scale from 0 (low fatigue) to 6 (high fatigue), except that the sleepiness question has ten levels. In addition, morning assessments also focused on their perceived sleep quality, while daytime sleepiness was measured three times daily. For further details, refer to Multimedia Supplementary Appendix B. These Patient PROs were collected via the SMA [37].

#### 2.2.3 Data preprocessing and feature extraction

For the PROs data, after inspection and correction for potential 12-h time shifts, the data were aggregated daily by taking the maximum symptom score over the day. Seven features related to nightly sleep, daytime sleepiness, and fatigue were then estimated (see Multimedia Supplementary Appendix C for the full list of PRO features).

For the sleep tracker data, invalid entries (considered outliers) were removed first. Sleep onset and wake-up times were extracted, and any daytime recordings were excluded. Daytime recordings were defined as those where sleep onset occurred after 6 a.m. with a wake-up time on the same day. Additionally, nighttime recordings shorter than two hours were discarded, with all excluded data classified as outliers. The remaining valid sleep data were analyzed further.

From these hypnograms, features characterizing each night’s sleep were first extracted and used for association with PRO analysis. Additionally, as all three sleep trackers also provide detailed heart rate and respiratory rate information, nightly physiological features were then extracted and combined with the nightly sleep data to be primarily utilized in the development of machine learning models. As a summary, all aforementioned features, i.e., 24 features for ZKONE, 28 for BedSensor, and 21 for DREEM 2 per night per participant, are listed in Supplementary Appendix D.

### 2.3 Feasibility study data coverage and quality

To assess the data coverage collected from a sleep tracker during the FS, the number of nights with valid sleep data for each participant was divided by the total number of nights they were expected to use the device according to the study protocol. The average coverage across all participants was then calculated to determine the overall coverage for the sleep tracker.

Table 2 presents the patient demographics, including sex, age, and Body Mass Index (BMI), as well as the number of nights/days for which both valid sleep information from trackers and the corresponding PRO scores for the following day are available. The total number of participants with relevant data is 134, encompassing 3,062 days/nights of data.

**Table 2:** FS: sleep-related demographics.

Cohort group	Cohort	Site	N	Female	Male	Testing days	Age, Mean (STD)	Age, Range	BMI, Mean (STD)
Healthy	Healthy	E, G, K, N	38	20	18	880	49.1 (16.3)	21–77	26.5 (4.9)
NDD	HD	G, K	12	6	6	242	44.6 (9.7)	30–60	26.4 (6.7)
	PD	K	18	7	11	357	62.1 (12.2)	37–80	24.3 (2.6)
IMID	IBD	E	15	8	7	305	37.7 (11.2)	22–55	24.4 (3.3)
	PSS	N	18	16	2	452	61.2 (12.6)	37–82	26.2 (4.0)
	RA	K, N	16	13	3	401	63.7 (11.9)	39–79	29.9 (8.0)
	SLE	K, N	17	17	0	425	49.0 (13.6)	31–80	26.3 (5.9)
Total	7 cohorts	4 sites	134	87 (64.9%)	47 (35.1%)	3,062	52.8 (15.8)	21–82	26.4 (5.5)

PD, Parkinson’s disease; HD, Huntington’s disease; IBD, Inflammatory bowel disease; PSS, primary Sjogren’s syndrome; RA, rheumatoid arthritis; SLE, systemic lupus erythematosus. Participating Centers: K, Universitätsklinikum Schleswig-Holstein Kiel; N, Newcastle University; E, Erasmus University Medical Centre Rotterdam; G, George-Huntington-Institute; BMI, Body Mass Index.

**Figure 2:**
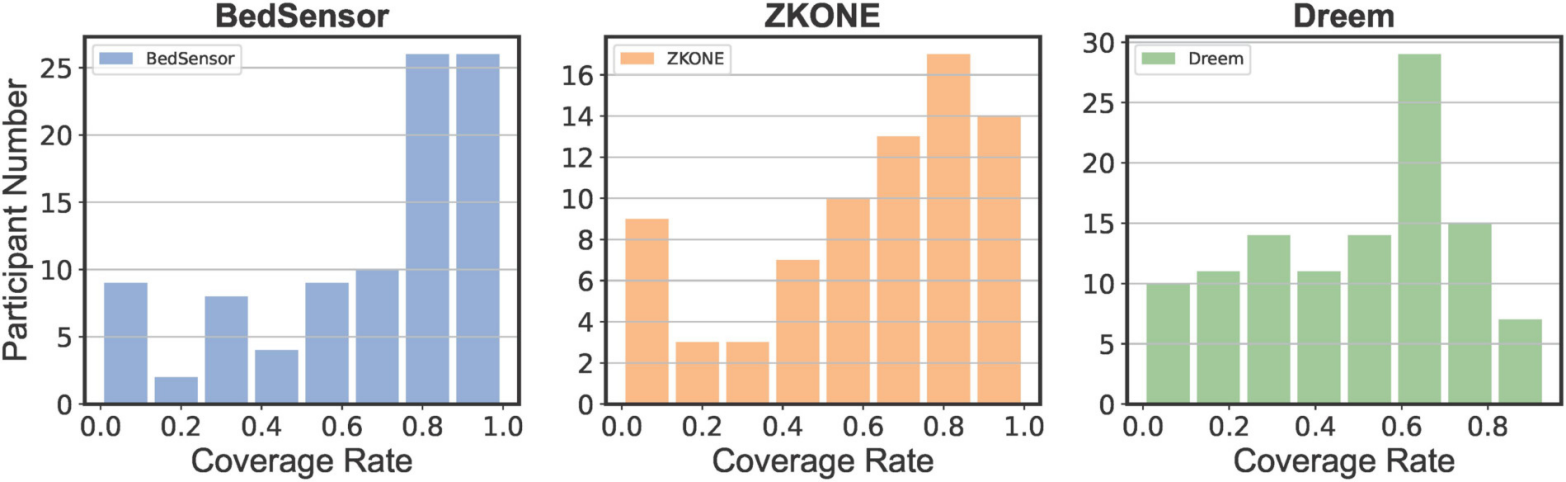
Histograms of sleep tracker’s data coverage rates.

By kicking out outlier recordings with a sleep duration of less than two hours, Figure 2 shows the distribution of participants’ coverage ratios, representing the proportion of nights with valid data over tested nights for each sleep tracker. Notably, BedSensor shows the highest performance, with the majority of participants achieving over 70% coverage rates. This superior performance is likely due to BedSensor’s reliable force-induction sensory system positioned under the mattress and its stable physical data transmission method. In contrast, ZKONE and DREEM 2 face challenges, primarily due to unstable Wi-Fi connections and user comfort issues, respectively.

Additionally, Table 3 provides a detailed data quality assessment for the three sleep trackers. BedSensor demonstrates superior performance, with the highest median coverage rate of 78.6%. In terms of outlier rate comparison, ZKONE excels, showing zero outliers for 77 subjects.

**Table 3:** Summary of sleep tracker data quality.

Device	ZKONE			BedSensor			DREEM2		
Number of participants	77			98			114		
Total nights	1334			1828			776		
Med. coverage rate	69.0%			78.6%			53.3%		
Number of participants per cohort	NDD	IMID	Healthy	NDD	IMID	Healthy	NDD	IMID	Healthy
	22	30	25	25	45	28	22	57	35
Med. coverage rate per cohort	65.3%	70.9%	71.4%	65.5%	80.6%	78.7%	36.7%	55.6%	48.3%

#### 2.3.1 Statistical analysis

Repeated measures correlation (RMC) [38] was used to quantify the associations between PROs and the measures extracted from each sleep device. The analysis was first conducted for all participants together, followed by separate analyses for healthy volunteers, NDD, and IMID cohorts. This approach aims to identify specific feature associations or cohort effects within these groups. After computing the correlations, only correlation coefficients *r*-values associated with *p*-values less than 0.05 were considered statistically significant. To control for multiple comparisons in the pairwise repeated-measures correlation (RMC) analyses, the Benjamini–Hochberg procedure was applied to adjust the *p*-values. The statistically significant associations following this correction were visualised using correlation heatmaps. Group differences were assessed using the Mann–Whitney U test, and the results were presented through box plot visualisations.

#### 2.3.2 Machine learning modeling and feature importance

Machine learning models were developed to classify high vs. low levels of physical fatigue, mental fatigue, and daytime sleepiness on the day following a night’s sleep using sleep and physiological features obtained from sleep trackers. To define the binary outcomes, the mean and standard deviation across all participants and recordings were calculated for each outcome variable. A threshold of one standard deviation above the mean was used to identify high values, while values below this threshold were classified as low [39, 40]. This approach was chosen for cross-cohort comparability but introduces a known interpretability constraint in heterogeneous disease populations.

Feature preprocessing and model development were designed to minimize potential sources of bias and data leakage. Feature selection was first performed based on variability: sleep-tracker features were evaluated using their interquartile ranges, and features with no variability were excluded to avoid introducing non-informative predictors. During model training, the remaining features were normalized using the Z-score method (the mean and standard deviation were calculated based on the training subset) to ensure comparable scaling across variables.

To prevent information leakage between training and testing data, all preprocessing steps were performed exclusively within the training data of each fold. Specifically, normalization parameters were computed using only the training subset within each leave-one-subject-out cross-validation (LOSO-CV) fold and then applied to the corresponding test subject. Missing values were imputed in a hierarchical manner using training data only, first using the subject-level mean, followed by subgroup-level means, and finally population-level means when prior estimates were unavailable.

Model evaluation was conducted using LOSO-CV to mitigate subject-level dependency and ensure that data from the same participant did not appear in both training and testing sets. This approach provides a more realistic estimate of generalization performance for unseen individuals. Three classifiers were evaluated: Random Forest (RF), Multilayer Perceptron (MLP), and regularized logistic regression (LASSO). Hyperparameters for each model were optimized using grid search within a nested cross-validation framework to reduce the risk of overfitting and optimistic bias in performance estimates (refer to Supplementary Appendix F). Each experiment was conducted five times. Several post-hoc analyses were conducted to assess potential biases arising from class imbalance, limited sample sizes, and cross-cohort data enrichment.

The primary performance metric reported throughout this study is the Area Under the Receiver Operating Characteristic Curve (ROC AUC, hereafter AUC), computed as the area under the full ROC curve across all classification thresholds. Given substantial class imbalance in some cohorts (e.g., high-fatigue class prevalence of 0.18–0.20 in the Healthy cohort), results were supplemented with Precision–Recall AUC (PRAUC) to provide a more informative assessment of minority-class performance. Uncertainty was quantified using 95% confidence intervals derived from 1,000 bootstrap iterations with subject-level resampling applied consistently across experimental runs. Separately, an optimal classification threshold $c^{*}$ was identified for each model by evaluating Youden’s index Supplementary Appendix F at every threshold c generated from the ROC curve, and optimal threshold was used solely to derive the sensitivity and specificity values reported in Table 5.

To investigate whether augmenting cohort-specific training sets with data from other cohorts could improve predictive performance, an enrichment analysis was performed. For each best-performing model configuration, baseline performance (trained on the target cohort only) was compared against enriched training conditions: (a) trained on all non-healthy-volunteer cohorts, and (b) trained on the full combined dataset including all cohorts. The change in AUC (ΔAUC) was computed as paired differences across matched experiment runs. To assess whether training on enriched cohorts (i.e., pooling data from multiple disease groups) significantly improved classification performance compared to training on the target cohort alone, we employed a subject-level permutation test. This approach respects the clustered structure of our data, where each subject contributes multiple observations (nights). Under the null hypothesis of no difference between models, we randomly swapped each subject’s predicted probabilities between the baseline and enriched models, then recomputed the difference in AUC (ΔAUC). This permutation was repeated 1,000 times to generate a null distribution. The two-sided p-value was calculated as the proportion of permuted (ΔAUC) values greater than or equal to the observed (ΔAUC). For directional hypothesis testing (i.e., whether enrichment improves AUC), we report the one-sided p-value, with significance assessed at α=0.05.

For transparency, the number of subjects and total samples (recording nights) were reported for each experimental configuration. This allows the reader to assess the reliability of performance estimates in light of the available data. Configurations with small subject counts (e.g., NDD cohort with n=29 subjects) or extreme class imbalance are explicitly flagged as requiring cautious interpretation, as such small samples yield high variance in cross-validated performance estimates.

In total, 135 models were developed across outcome variables (n=3: physical fatigue, mental fatigue, and daytime sleepiness), sleep trackers (n=3), participant cohorts (n=5: all participants, healthy individuals, IMIDs, NDDs, and IMIDs combined with NDDs), and classifiers (RF, MLP, and LASSO). Model performance was evaluated using LOSO-CV (see Supplementary Appendix F for performance metrics). To interpret model predictions and assess feature contributions, SHapley Additive exPlanations (SHAP) were applied. Features were ranked based on their contribution magnitude and visualized using beeswarm plots showing the ten most influential predictors.

## 3 Results

### 3.1 Validation of sleep trackers vs. polysomnography

**Figure 3:**
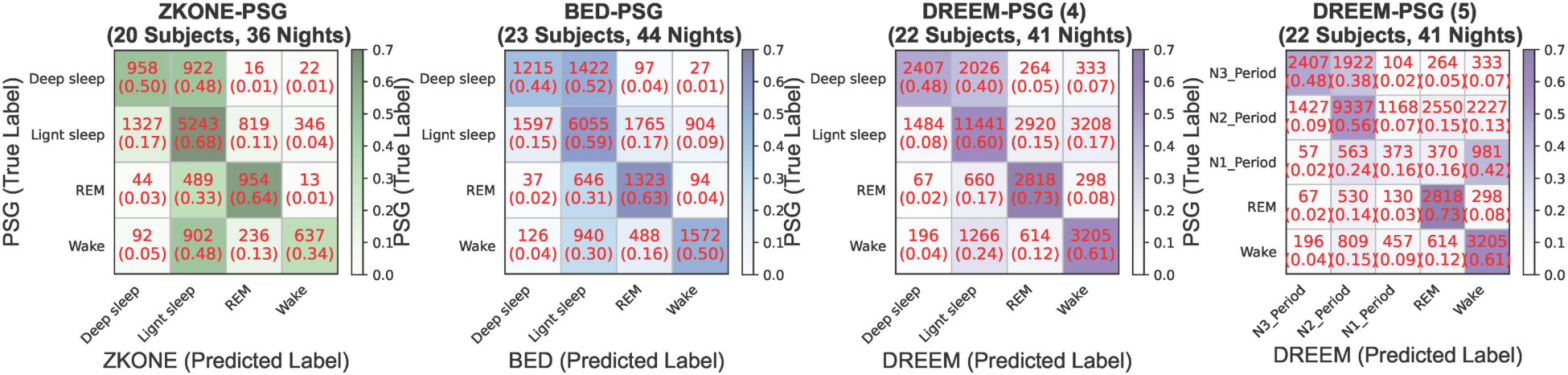
Confusion matrices of hypnogram comparisons between three sleep trackers (ZKONE, BedSensor, DREEM 2) and PSG. Numbers in each cell correspond to the number of hypnogram epochs overlapping 30 s between the tracker and PSG. The number in brackets in each cell indicates the corresponding ratio to the total number of epochs for the considered sleep stage. BED = BedSensor. DREEM-PSG (4-stage) refers to the comparison where DREEM 2’s detailed sleep stages, “deep sleep”, “light sleep”, “REM”, and “wake” were used. In contrast, DREEM-PSG (5-stage) represents the case where DREEM 2’s sleep stages were grouped into broader categories: “N1, N2, N3, REM, and wake”.

**Figure 4:**
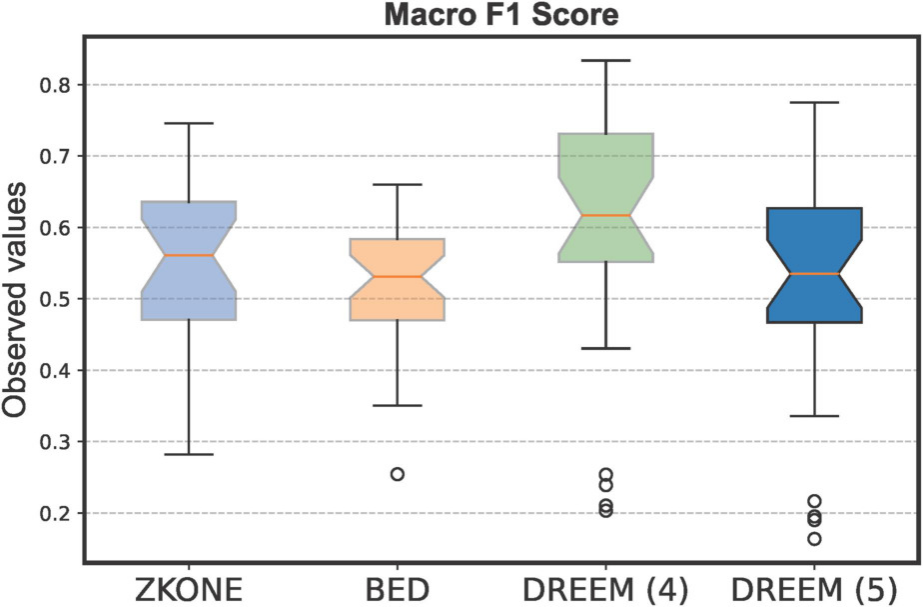
Comparison of the macro F1 scores for agreement between the three sleep tracking devices and PSG. BED = BedSensor. DREEM-PSG (4) refers to the comparison where DREEM 2’s detailed sleep stages, “deep sleep”, “light sleep”, “REM”, and “wake” were used. In contrast, DREEM-PSG (5) represents the case where DREEM 2’s sleep stages were grouped into broader categories: “N1, N2, N3, REM, and wake”.

Figure 3 shows confusion matrices for the validation of ZKONE, BedSensor, DREEM 2 vs. PSG. In general, all three devices were poor at detecting “deep sleep”, but better at detecting “light sleep” and REM. Notably, among all trackers, DREEM 2 is able to achieve a higher true positive rate. In addition, Figure 4 displays the agreement between sleep trackers and PSG by using the macro F1 score. The result shows that the DREEM 2 headband can reach the highest macro F1 score with a median of 0.62. PSG study further demonstrates the feasibility of applying these devices to patients, suggesting that the sleep-tracking approaches involved in this study retain a reasonable level of generalizability when used in IMID and NDD cohorts.

### 3.2 Association between sleep tracker-derived and PRO-derived characteristics: all participants

Table 4 summarizes the outcomes of RMC analysis [38] between sleep tracker-derived features and PRO metrics across all participants. For clarity, PRO features are classified into two groups, fatigue (i.e., physical and mental fatigue) and sleep (i.e., sleepiness, sleep duration, sleep quality, fall asleep time, and wake duration). The table displays the range of absolute correlation coefficients (p<0.05) and reports the top association per PRO group. After Benjamini–Hochberg correction for multiple comparisons, no statistically significant associations survived between sleep tracker features and fatigue-related PRO metrics (physical or mental fatigue). The remaining significant associations are confined to sleep-related PRO metrics, which tend to show stronger correlations with sleep time measurements.

**Table 4:** Summary of top association between sleep tracker-derived features and PRO metrics per PRO group, when all participants are considered together.

Device	PRO group	|r| Range	Top association (PRO feature vs. tracker)
BedSensor	Sleep	[0.09, 0.48]	Sleep Duration vs. TST
ZKONE	Sleep	[0.11, 0.35]	Sleep Duration vs. TST
DREEM 2	Sleep	[0.14, 0.53]	Sleep Duration vs. TST

TST, Total Sleep Time derived from sleep tracker.

Specifically, the sleep duration obtained from PROs shows the highest correlation (a positive r value of 0.53) with the Total Sleep Time (TST) estimated by the DREEM 2 headband. This indicates that the DREEM 2 device offers greater consistency in measuring TST compared to subjective assessments.

### 3.3 Association between sleep tracker-derived and PRO-derived characteristics: cohort effects

**Figure 5:**
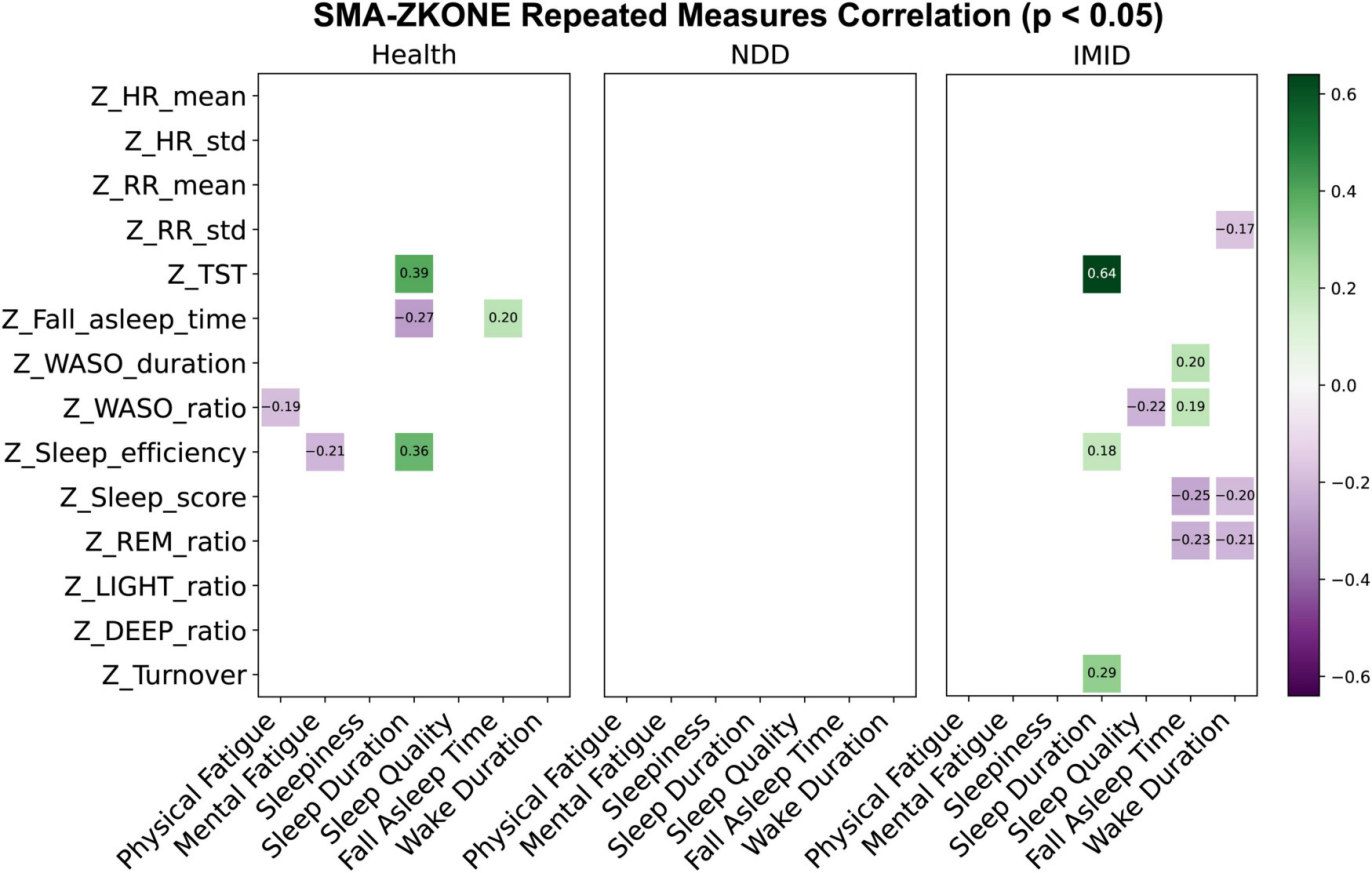
Heatmap of repeated measures correlation between PRO’s and ZKONE sleep tracker’s metrics. Significant correlations (p<0.05) are color-coded according to the strength (*r*-value) of their association. The interpretation of the features provided by ZKONE could be found in Multimedia Table D1 in Supplementary Appendix D.

Figures 5 through 7 display the repeated measures correlation heatmaps for the Healthy, NDD, and IMID cohorts, showing the associations between PROs metrics and sleep features derived from ZKONE, BedSensor, and DREEM trackers, respectively. These figures highlight that subjective sleep duration, as reported by all cohorts, exhibits the highest correlation with the TST estimates from these three trackers.

**Figure 6:**
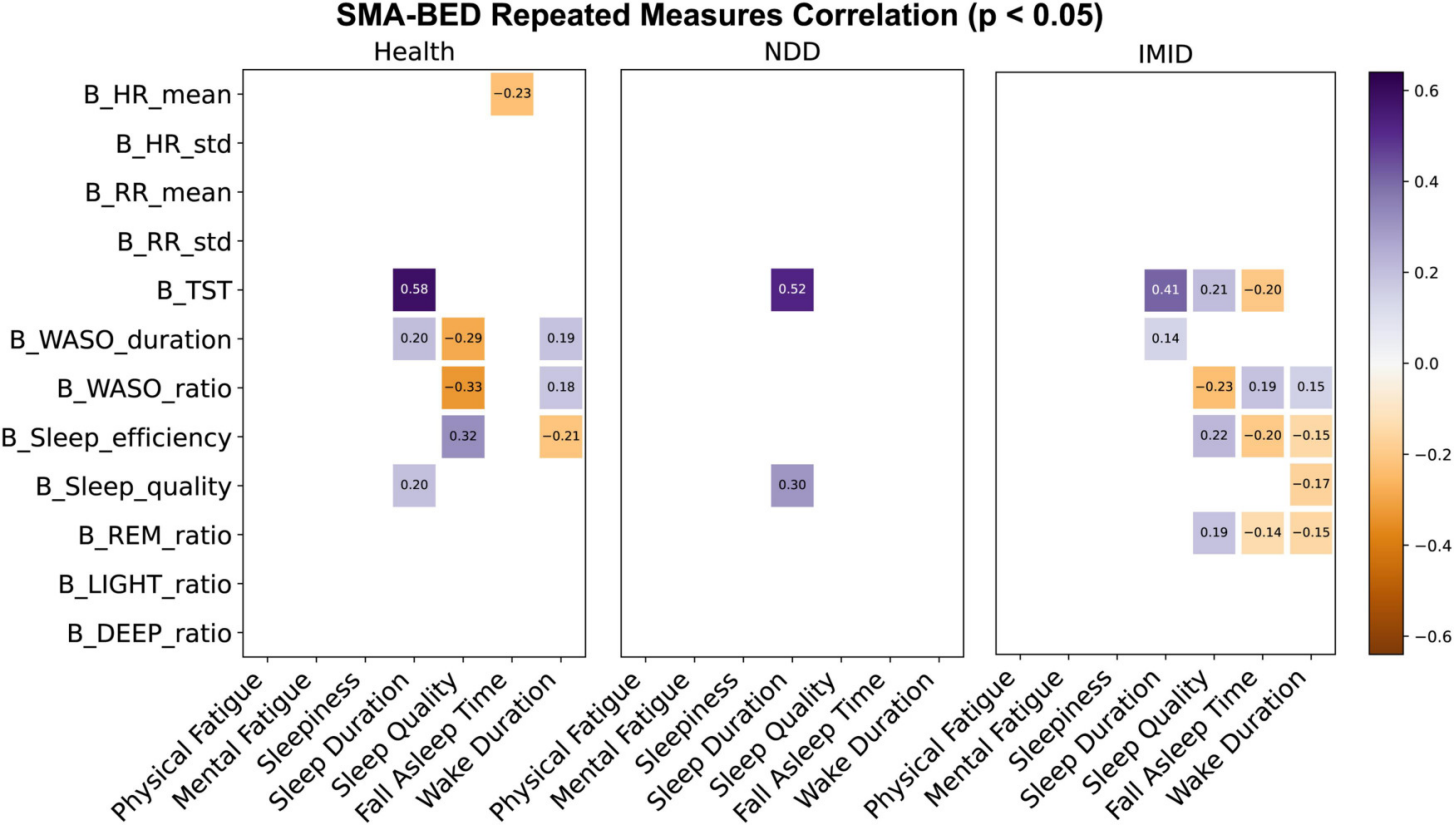
Heatmap of repeated measures correlation between PRO’s and BedSensor sleep tracker’s metrics. Significant correlations (p<0.05) are color-coded according to the strength (*r*-value) of their association. The interpretation of the features provided by BedSensor could be found in Multimedia Table D2 in Supplementary Appendix D.

**Figure 7:**
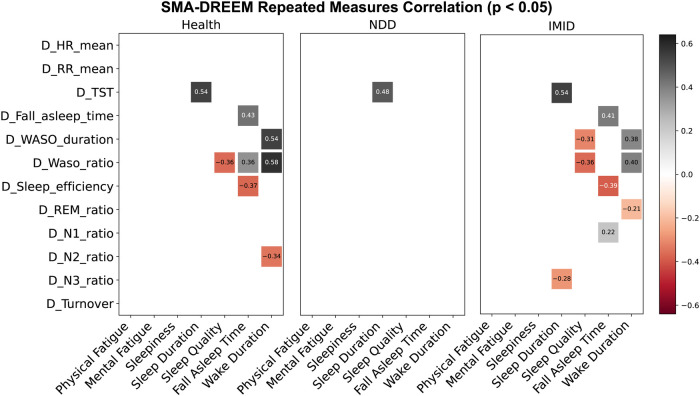
Heatmap of repeated measures correlation between PRO’s and DREEM 2 headband’s metrics. Significant correlations (p<0.05) are color-coded according to the strength (*r*-value) of their association. The interpretation of the features provided by DREEM 2 could be found in Multimedia Table D3 in Supplementary Appendix D.

Notably, for the healthy cohort, PRO-derived wake duration shows a high positive correlation (r=0.58) with DREEM’s wake after sleep onset (WASO) ratio. For the IMID cohort, DREEM’s sleep efficiency estimation and the time participants fall asleep is highly associated with PRO-derived fall asleep time, with correlation values of −0.39 and 0.41, respectively. For the NDD cohort, BedSensor’s sleep quality shows a distinct correlation (r=0.3) with PRO-derived sleep duration.

Regarding fatigue related PRO metrics, after Benjamini-Hochberg correction, ZKONE’s sleep efficiency feature shows a nominally significant association (r=−0.21) with self-reported mental fatigue in the healthy group only. No significant associations between tracker-derived sleep features and fatigue PROs survived correction in the NDD or IMID cohorts.

### 3.4 Machine learning modelling

Table 5 summarises the LOSO cross-validation performance for predicting next-day fatigue and sleepiness from preceding-night sleep tracker data. In the healthy cohort (N=38), the BedSensor with LR combination achieved the strongest performance for physical fatigue (AUC = 0.75), with minimal change upon training data enrichment, whereas mental fatigue prediction improved from 0.58 to 0.66 when all participants were included. Training data enrichment yielded the most gains in smaller patient cohorts: in the NDD cohort (N=29), physical fatigue AUC increased from 0.17 to 0.62 (ΔAUC =+0.45, p=0.12) when trained on all participants, and in the IMID cohort (N=64), enrichment with patient participants improved AUC from 0.51 to 0.63 (ΔAUC =+0.12, p=0.04), albeit with a trade-off between high sensitivity (0.85) and lower specificity (0.44). Sleepiness prediction in the NDD cohort showed only modest, non-significant gains from enrichment (ΔAUC =+0.16). Overall, these results suggest that cross-cohort pooling can improve predictive performance, particularly for smaller patient groups with limited within-group variance.

**Table 5:** Summary of leave-one-participant-out performance results for predicting next-day low vs. high subjective fatigue and sleepiness using sleep tracker characteristics from the preceding night, by specific cohorts.

Prediction	Device-Model	Cohort (Test)	AUC			Sensitivity	PRAUC	Specificity
			Trained on individual group	Trained on No-Health participants	Trained on all participants			
Physical Fatigue	beds-LR (Ridge)	Healthy N=38	0.75 [0.34, 0.90]	–	0.75 [0.48, 0.92]	0.62 [0.23, 0.90]	0.51 [0.10, 0.86]	0.76 [0.63, 0.88]
Mental Fatigue	beds-LR (Ridge)	Healthy N=38	0.58 [0.31, 0.80]	–	0.66 [0.40, 0.81]	0.66 [0.28, 0.85]	0.32 [0.08, 0.64]	0.69 [0.46, 0.85]
Physical Fatigue	dreem-RF	NDD N=29	0.17 [0.06, 0.28]	0.50 [0.19, 0.70] +0.33 [0.11, 0.67] N=97, p=0.25	0.62 [0.28, 0.84] +0.45 [0.17, 0.66] N=134, p=0.12	0.20 [−0.00, 0.33]	0.15 [0.03, 0.29]	0.74 [0.33, 0.98]
Physical Fatigue	zkone-RF	IMID N=64	0.51 [0.38, 0.64]	0.63 [0.50, 0.75] +0.12 [−0.03, 0.23] N=93 p=0.04	0.59 [0.41, 0.75] +0.08 [−0.12, 0.26] N=130			
p=0.27	0.85 [0.66, 0.91]	0.42 [0.20, 0.71]	0.44 [0.27, 0.63]					
Sleepiness	dreem-RF	NDD N=29	0.50 [0.26, 0.73]	0.66 [0.37, 0.91] +0.16 [−0.16, 0.39] N=97 p=0.29	0.54 [0.37, 0.86] +0.04 [−0.10, 0.44] N=134 p=0.38	0.33 [0.00, 0.80]	0.40 [0.05, 0.83]	0.89 [0.75, 0.98]

Results are shown for cohort-specific training data as well as enriched training sets (including all patients or all participants). Top-performing device-model combinations were identified based on test data results, with AUC improvements noted in brackets following the enrichment of the training dataset. RF - random forest, LR - LASSO logistic regression.The performance improvement achieved through training data enrichment is reported, and p value is reported for significant improvements. Note: Participants exhibiting no variability in either features or responses were excluded.

### 3.5 Feature importance

**Figure 8:**
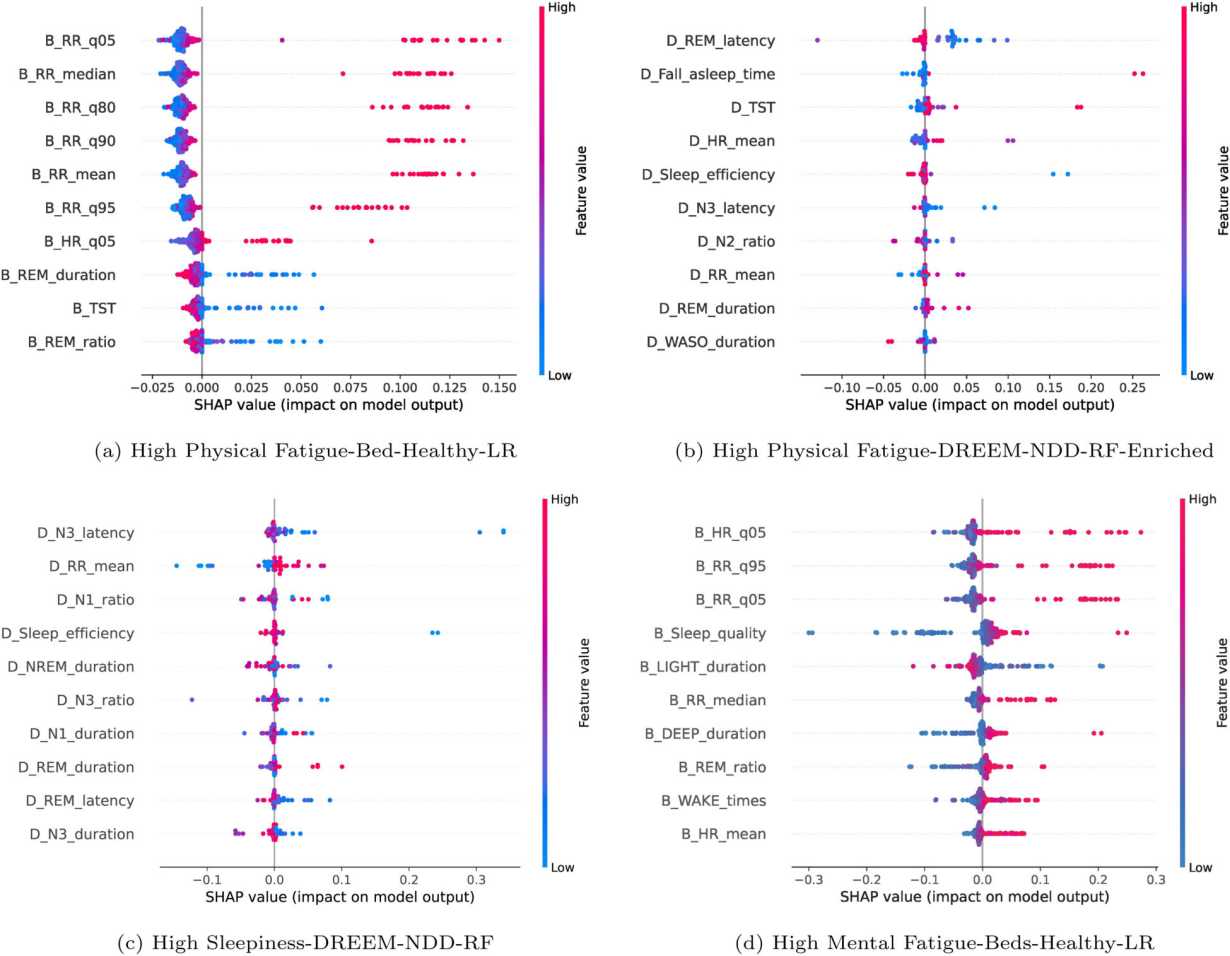
Importance of sleep tracker features in predicting next-day fatigue. Features are ordered by importance from top to bottom. The horizontal axis shows the changes in log odds. The long tails highlight the heightened importance of specific features for individual patients. Each point represents a model’s prediction for a specific night-day combination. The color-coding of dots—blue for low and pink for high feature values—alongside their distribution on the horizontal axis, which quantifies the impact magnitude, reveals the importance and directionality of each feature’s effect. Note: Results in panels **(b–d)** are based on models (highest AUC) trained on enriched data. **(a)** High Physical Fatigue-Bed-Healthy-LR, **(b)** High Physical Fatigue-DREEM-NDD-RF-Enriched, **(c)** High Sleepiness-DREEM-NDD-RF, **(d)** High Mental Fatigue-Beds-Healthy-LR.

The global feature importance scores were computed using SHapley Additive exPlanations (SHAP) values from the top-performing models. In Figure 8, each dot represents a SHAP value for a feature in a single instance of predicting next-day PRO metrics, indicating the feature’s impact on the model output.

In healthy controls (see Figure 8a), features related to respiratory rates (RR), such as the median and 5th, 80th, 90th, and 95th percentiles, are prominent in their contribution to the prediction of next-day physical fatigue using data from BedSensors are predictors (Ridge logistic regression, AUC = 0.75). Higher values of these RR metrics (indicated by pink dots) substantially increase the log odds of predicting high levels of physical fatigue. Conversely, lower feature values (represented by blue dots) are associated with decreased log odds of high fatigue, suggesting that lower nightly respiratory rates may predict lesser fatigue the following day.

Figures 8b–d show SHAP-derived feature importance from the highest-performing enriched models, where training data were pooled across cohorts. Because these models learned from both between-person and cross-cohort variation, the resulting feature rankings here is interpreted as exploratory and hypothesis-generating observations rather than evidence of disease-specific mechanistic predictors of fatigue. Figure 8b presents the most important features for predicting next-day physical fatigue in the NDD cohort using data from DREEM 2 devices (Random Forest, AUC = 0.62). Key features include the latency of the first episode of REM sleep, time to fall asleep, Total Sleep Time, and nightly mean HR. The most influential feature identified is the latency of REM sleep, where shorter latency is associated with higher log odds of predicting next-day high physical fatigue. However, this feature and total sleep time alone did not significantly differentiate the groups, suggesting that interactions with other features may improve predictive accuracy in the NDD population.

Similarly, a negative contribution is observed for the sleep efficiency metric, where lower sleep efficiency (indicating more waking during sleep) contributes to higher next-day fatigue levels. Metrics such as the latency of the first episode of the N3 sleep stage and the ratio of N2 to Total Sleep Time also show negative SHAP values. These findings illustrate that higher sleep efficiency and quicker transitions to deeper sleep stages are associated with lower fatigue levels the next day. This insight underscores the protective role of regulated sleep in mitigating fatigue symptoms in patients with neurodegenerative diseases. Figure 8c illustrates the feature importance for predicting next-day mental fatigue utilizing BedSensor data in healthy individuals using Ridge logistic regression (AUC = 0.66). The top ten features, such as sleep quality, the 95th percentile of RR, and the 5th percentiles of both HR and RR, stand out due to their distribution and density across the plot.

**Figure 9:**
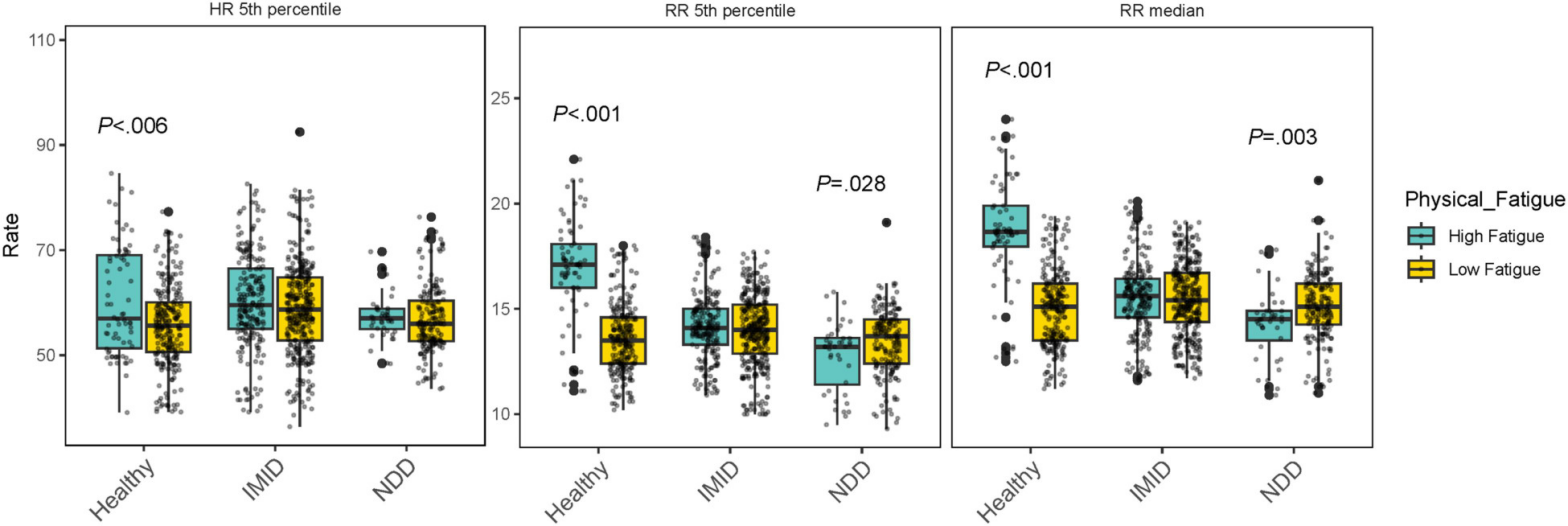
Boxplots showing duration differences in HR 5th percentile, RR 5th percentile, and RR median across groups by Physical Fatigue levels (High and Low Fatigue). Statistically significant p-values indicate with-in group differences, with high physical fatigue individuals generally displaying higher respiratory rate and HR in Healthy participants compared to low fatigue counterparts.

We further investigated those features which were reported to be the most important. The sleep and physiological predictors are shown in Figure 9. For healthy individuals, high physical fatigue experienced during the day is typically preceded by elevated heart rate (p=0.006) and respiratory rate (p≤0.001) during the previous night’s sleep. However, this pattern does not occur in individuals with IMID. Conversely, for individuals with NDD, the median respiratory rate shows the opposite pattern (p=0.003).

**Figure 10:**
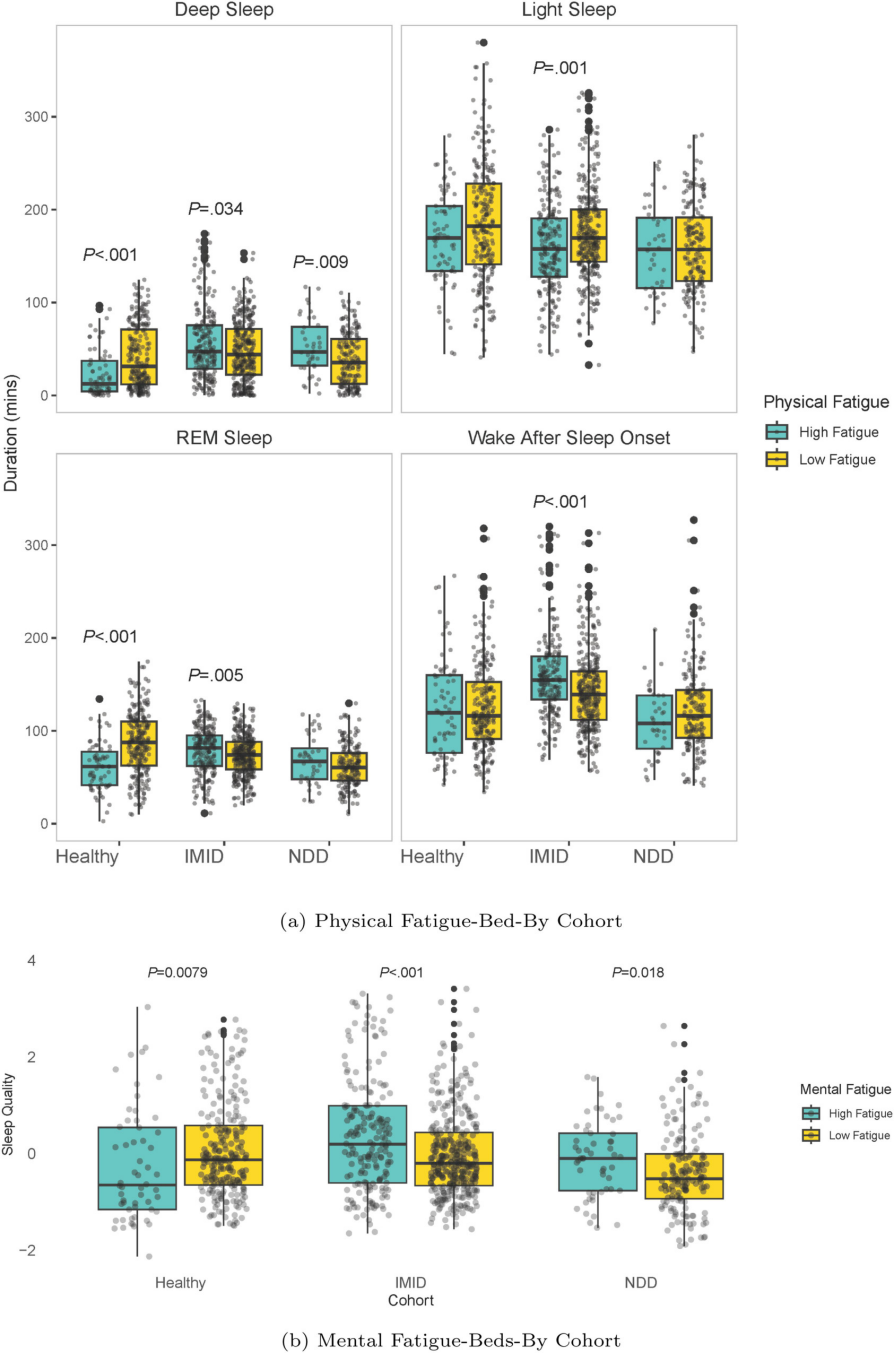
Sleep-related feature comparison between groups with low and high levels of fatigue: **(a)** Comparison of sleep stages derived from BedSensor across different cohorts and physical fatigue levels; **(b)** The sleep quality metric measured from BedSensors displayed for each cohort (mental fatigue). **(a)** Physical Fatigue-Bed-By Cohort, **(b)** Mental Fatigue-Beds-By Cohort.

Figure 10a presents within-cohort comparisons of sleep-related metrics. In healthy individuals, high daytime physical fatigue is linked to significantly reduced deep and REM sleep duration (both predictors, p≤0.001). For IMIDs, this relationship is reversed with both increases in deep (p=0.034) and REM sleep duration (p=0.005). The high physical fatigue IMID patients also have significantly reduced light sleep (p=0.001) and increased WASO (p≤0.001). Among individuals with NDDs, high physical fatigue is linked to a significant(p≤0.009) increase in deep sleep duration.

For low physical fatigue, IMID patients exhibited significantly longer WASO durations compared to both NDD patients and healthy participants (both comparisons, p≤0.001). Regarding REM sleep, healthy individuals had longer durations than both IMID and NDD patients (both comparisons, p≤0.001), while IMID patients showed longer REM durations than NDD patients (p≤0.001). For light sleep, both healthy and IMID participants displayed longer durations than NDDs (p≤0.001, respectively). Overall, NDD patients slept the least amount of time than IMID and healthy participants, while IMID patients experienced significantly greater sleep disruption (WASOs) and more deep sleep compared to the other groups. These findings suggest that pathological factors may play a larger role than physiological ones in low physical fatigue.

We have to highlight that, for Figures 9 and 10, the thresholds for healthy individuals were largely consistent with the global thresholds, but the population-specific thresholds for IMIDs and NDDs differed from the global thresholds. These results indicate differences in these measures at the global thresholds rather than differences between high and low physical fatigue within a group. The interpretation of these results do not imply the digital biomarkers for predicting disease-specific fatigue.

## 4 Discussion

Fatigue is a pervasive and difficult-to-manage symptom across many chronic conditions, yet objective tools to monitor and anticipate fatigue in daily life remain limited. Accurate prediction of next-day fatigue could enable more proactive symptom management, allowing patients and clinicians to adjust daily activities, optimize treatment timing, or implement targeted interventions before severe fatigue occurs. Such predictive capability could also support remote monitoring and personalized care strategies, particularly for individuals with chronic conditions where fatigue fluctuates substantially over time.

Our findings demonstrate the feasibility of using nightly sleep physiology captured by wearable sleep trackers to evaluate next-day fatigue, supporting the potential of these devices as scalable digital device for fatigue monitoring in home settings. Importantly, the observed differences between healthy individuals and clinical cohorts highlight that the relationship between sleep and fatigue may vary across diseases, underscoring the need for disease-specific approaches when developing digital fatigue management strategies.

### 4.1 Sleep tracker validation

We have validated three sleep trackers, each representing different technologies for sleep assessment: BedSensor, ZKONE, and DREEM 2. While all three trackers previously demonstrated good validity in healthy populations [41–44], our results showed lower correspondence with polysomnography (PSG) in a population with NDD and IMID diseases. This discrepancy suggests that the algorithms behind these technologies, which were trained on healthy populations, may not generalize well to populations with diseases. This underscores the importance of validating sleep technology in relevant populations before its use in clinical trials, as emphasized by regulatory agencies like the FDA [45].

The performance differences between healthy individuals and those with disease may also explain the disparity in classification performance when predicting next-day fatigue and sleepiness in the healthy cohort compared to the patient cohorts in our study. Despite the lower validation results in NDD and IMID populations, the features obtained from sleep trackers in these disease populations may still be adequate for specific research questions. Therefore, we continued with these devices for an FS to explore whether and how nightly sleep affects next-day fatigue and sleepiness.

### 4.2 Insights from the analysis of correlations between sleep features and PRO scores

Inter-subject response preference was evident when participants reported their fatigue and sleepiness PROs. Due to differences in individual perceptions, people responded differently when selecting specific numbers to describe their fatigue and sleepiness feelings. Some individuals tended to select higher scores to rate their fatigue and sleepiness baselines, while others chose lower values, even though they may have similar fatigue/sleepiness symptoms. Across individual raw responses, floor and ceiling effects were minimal for all measures. The largest observed effect was a floor effect of 6.1% for Sleepiness, with all other floor and ceiling proportions remaining below this level and well under the conventional 15% threshold [46]. Daily aggregation of responses likewise produced low boundary effects. For mean aggregation, the maximum observed effect was a 4.3% floor effect (Sleepiness); for median aggregation, a 5.0% floor effect (Sleepiness); and for maximum aggregation, a 4.3% floor effect (Sleepiness). Ceiling effects were consistently smaller than floor effects across aggregation methods.

We then employed RMC with Benjamini–Hochberg correction to examine associations between fatigue/sleepiness and digital sleep features within each cohort. The analysis of the correlation results between sleep tracker features and participants’ subjective feelings revealed that the largest effect size is observed between objective and subjective assessments of nightly sleep. As expected, the highest repeated measures correlation is between the total sleep time derived from devices and the subjective assessment of sleep duration. This alignment between objective measurements and individuals’ impressions of their sleep is crucial for our subsequent discussion on fatigue and daytime sleepiness.

In contrast, associations between sleep features and PROs of fatigue or sleepiness were generally weaker, consistent with fatigue and sleepiness reflecting a mixture of physiological state and subjective perception. Digital health technologies may be better suited to capturing *changes* in these states within an individual rather than absolute symptom levels across individuals. Motivated by this, the ongoing IDEA-FAST clinical observational study has adjusted fatigue/sleepiness questionnaires to include more concrete, change-focused questions (e.g., how fatigue and sleepiness differ from the previous day).

### 4.3 Insights from machine learning modeling

#### 4.3.1 Physical fatigue

The best model performance in this study was achieved for predicting low vs. high physical fatigue in healthy adults (AUC = 0.75, sensitivity = 0.62, specificity = 0.76), with cardiorespiratory features during sleep among the most important predictors. These results suggest that nightly sleep physiology contributes meaningfully to next-day physical fatigue in healthy participants. In contrast, predictive performance was lower in chronic disease cohorts, which may reflect both (i) reduced device validity in these populations and (ii) the likelihood that fatigue in NDD and IMID groups is influenced more strongly by non-sleep-related factors (i.e., pathological fatigue). This interpretation aligns with patient reports that fatigue can be unrelated or only minimally related to sleep [3].

Within the NDD cohort, total sleep time emerged as a key predictor of physical fatigue using DREEM 2, both locally (via SHAP) and globally (via correlation), with greater physical fatigue associated with longer sleep duration. For ZKONE, we observed a negative correlation between WASO and physical fatigue in healthy adults; however, the Lasso model did not identify ZKONE features as useful for next-day fatigue prediction. Notably, we found no correlation between ZKONE WASO and participants’ self-reported wake duration or sleep quality. The confusion matrix (Figure 3) indicated that ZKONE overestimated WASO by classifying more light-sleep epochs as wakefulness, which may reduce its utility for fatigue prediction. Additionally, turnover was negatively correlated with physical fatigue in the NDD group, though it did not appear among the top predictors, which may reflect limited sample size and model stability.

The prominence of cardiorespiratory predictors suggests that higher physical fatigue is associated with altered breathing patterns during sleep. Although sleep apnea is known to relate to fatigue [47, 48], participants with diagnosed sleep apnea were *excluded* at recruitment to avoid confounding. Therefore, our results suggest that even in the absence of diagnosed sleep apnea, subtle deviations in sleep-related breathing and cardiorespiratory physiology may signal reduced restorative sleep and higher next-day fatigue.

Among the devices, DREEM 2 achieved an AUC >0.6 for predicting physical fatigue in NDD participants. This is consistent with our PSG validation results showing that DREEM 2 outputs were more closely aligned with PSG, plausibly because DREEM 2 incorporates multiple sensing modalities (brain activity, movement/position, breathing, and heart rate), whereas ZKONE and BedSensor rely predominantly on cardiorespiratory proxies for sleep staging.

#### 4.3.2 Mental fatigue

Prediction of high mental fatigue reached modest performance in healthy controls. Feature importance suggested an association between sleep quality and next-day mental fatigue, supporting the role of adequate sleep for cognitive functioning and mental health. In healthy adults, better nightly sleep quality was associated with lower mental fatigue the following day, although sleep metrics were not among the top cardiorespiratory predictors. In participants with chronic conditions (IMIDs and NDDs), model performance for mental fatigue was not satisfactory, despite statistically significant differences between low and high mental fatigue groups. Because sleep quality (BedSensor) is a complex, non-linear function (reflecting sleep stage composition, cycle structure, and wake periods) and is typically developed based on healthy adult data, the observed patterns in patient cohorts (e.g., increased sleep quality with higher physical fatigue) should be interpreted cautiously. Physiological predictors (e.g., the 5th percentile of heart rate and the 5th and 95th percentiles of respiratory rate) were associated with mental fatigue, suggesting overlapping physiological markers between mental and physical fatigue in healthy participants, and potentially distinct mechanisms in chronic disease cohorts.

#### 4.3.3 Sleepiness

In our attempt to predict daytime sleepiness using characteristics of the preceding night’s sleep, only a small number of experiments achieved satisfactory performance (AUC >0.6). This result did not appear in the same experimental and equipment settings related to fatigue. Deep sleep duration and mean respiratory rate emerged as the two most influential features for predicting high sleepiness. The results indicate that the predictive capacity of nightly sleep characteristics obtained from sleep trackers for sleepiness does not align with that for physical fatigue. This suggests that sleepiness and fatigue have distinct physiological signatures, particularly when assessed as separate constructs in questionnaires. Future research should also consider exploring circadian-related metrics to enhance the assessment of sleepiness.

### 4.4 Relation to prior work and other IDEA-FAST publications

Prior research has extensively explored domain knowledge-driven features, not necessarily related to sleep, derived from neurophysiological evaluations and self-assessment scales, in conjunction with machine learning and deep learning techniques for fatigue detection [31, 49–53]. A significant portion of the existing literature predominantly focuses on context-specific evaluations of “acute fatigue” within *healthy* adult cohorts, characterized by transient effects associated with *specific tasks* in work environments with tests consisting of task-oriented perceptual-motor adjustments (e.g., operating a professional machine) or monitor displays (e.g., office mental workload related tasks) [54–56].

In contrast, the IDEA-FAST study aims to monitor fatigue in *naturalistic settings* among *individuals with neurodegenerative and inflammatory diseases*. This approach is crucial for understanding the complex interplay between chronic health conditions and fatigue. Prevailing hypotheses suggest that this type of fatigue may be linked to factors such as diminished neurotransmitter levels, impaired energy production, inflammation, and psychological elements like stress, anxiety, and depression [3]. The fatigue we investigate in this study manifests through various descriptive parameters, including increased discomfort, diminished daily living capabilities, and a reduced propensity to respond to stimuli, generally accompanied by feelings of exhaustion [3, 57]. Specifically for healthy adults, our results indicate that night-time respiratory rate-related features are among the most important predictors of next-day physical fatigue levels.

Our findings are consistent with prior work suggesting that physiological signals can support fatigue prediction, and that cardiorespiratory measures are often informative. For example, in a study using the clinical-grade Everion™ device in 28 healthy participants, physical fatigue classification achieved precision of 0.7 and recall of 0.73 [51]. While study designs and fatigue definitions vary substantially across the literature [51–53], these comparisons collectively suggest that sleep-adjacent physiology provides useful information for predicting physical fatigue, particularly in healthy cohorts.

For the mental fatigue, comparable studies examining sleep-derived predictors of next-day are scarce. One controlled laboratory study [50] characterised mental fatigue in healthy participants using heart rate variability during fatigue-inducing cognitive tasks and reported an AUC of 0.84. Differences in context (acute experimentally induced fatigue vs. chronic day-to-day symptoms) and underlying mechanisms may explain the discrepancy, and the pathophysiology of chronic mental fatigue remains incompletely understood.

Within the IDEA-FAST feasibility study, other reports highlighted the predictive contribution of walking activity and gait features [58] and cardio fitness [2]. Considering these findings alongside the sleep-derived predictors presented here, a combined model integrating sleep, activity, gait, and fitness-related metrics is a strong candidate for improved performance and broader applicability.

### 4.5 Overall interpretation across cohorts

Overall, this study emphasizes the complex connections between physical fatigue, physiological factors, and sleep architecture across various groups. Objective sleep measures indicate that, for healthy individuals, sleep characterized by sufficient deep sleep for physical restoration, REM sleep, and normal respiratory and heart rates shows reduced physical fatigue the following day. In individuals with IMID, paradoxically, elevated physical fatigue on the following day has increases in the duration of deep and REM sleep from the prior night. Additionally, for IMID patients, high physical fatigue is linked to significantly increased WASO and reduced light sleep, which indicates greater sleep fragmentation. Light sleep supports physical restoration by regulating metabolic processes and preparing the body for transitions, while deep sleep focuses on intensive tissue repair, immune system strengthening, and energy replenishment.

For individuals with NDD, prior nights to low fatigue are marked by the least amount of sleep time when compared to both healthy and IMID cohorts, a finding that aligns with previous studies [59–61]. Individuals with NDD experience higher physical fatigue, reflected in a significant increase in deep sleep duration from the previous night, while changes in other sleep stages show no significant differences. The results align with self-reports from NDD patients, indicating that sleep may not be fully related to fatigue. Similarly, in IMID patients, an increase in REM and deep sleep could aggravate physical fatigue, as patients often report that their fatigue is minimally related to good sleep. Instead, targeting factors (e.g., pain and inflammatory state) that reduce WASO and increase light sleep may help lessen physical fatigue in IMID patients. However, this strategy may be less applicable to individuals within the NDD cohort.

## 5 Limitations and future work

A key strength of this work is that it represents the first evaluation combining (i) validation of wearable sleep devices against polysomnography (PSG) and (ii) assessment of the utility of device-derived sleep features for predicting next-day fatigue and sleepiness across healthy adults and two chronic disease cohorts. However, several limitations should be considered.

First, the generalisability of device-derived sleep metrics appears lower in neurological (NDD) and immune-mediated inflammatory disease (IMID) populations, as reflected by reduced agreement with PSG. In addition, PSG data were available only for a limited subset of participants and did not allow a systematic evaluation of device performance across all patient groups. These measurement limitations may propagate uncertainty into downstream association and prediction analyses. It is important to note that comprehensive PSG validation across diseases was beyond the scope of the IDEA-FAST study, which instead aimed to evaluate the feasibility of using state-of-the-art wearable devices that balance measurement accuracy, usability, cost, and patient acceptance for long-term real-world monitoring.

Second, the relatively small sample size within individual disease cohorts limits statistical power and the generalisability of the findings. Although more than 3,000 nights of sleep data were collected, subgroup analyses, particularly those involving device-specific metrics such as DREEM 2-derived sleep architecture features, were constrained by limited observations. For example, we observed that shorter latency to the first REM episode was associated with higher next-day physical fatigue, suggesting a potential role of sleep architecture in energy recovery. However, this relationship did not reach statistical significance, likely due to limited sample size. These findings highlight the limitations of relying on single sleep metrics and support the need for larger datasets and multivariate approaches that capture interactions among multiple physiological features.

Third, the relationships among sleep, fatigue, and sleepiness appear complex, multifactorial, and potentially non-linear. Regression models tested both with and without mixed effects, including random effects for demographic and cohort factors, did not yield meaningful associations. While repeated-measures correlation analyses account for inter-subject response preferences, the machine-learning models did not explicitly adjust for individual differences in scale usage. Including demographic or cohort indicators could potentially improve classification performance but might bias models toward static between-person differences rather than capturing day-to-day symptom variability; therefore, these variables were excluded from the primary predictive models.

Several additional methodological considerations may have affected model performance. Binary classification of patient-reported outcomes (PROs) may obscure clinically meaningful gradations in fatigue and sleepiness. Another limitation relates to the class definition and resulting imbalance. High fatigue was defined using a global threshold (≥1 SD above the overall sample mean), which may introduce bias toward the majority low-fatigue class. We also investigated whether the population-level threshold (mean + 1 SD computed across all participants) conflates between healthy and disease cohort differences, and applied cohort-specific thresholds (mean + 1 SD computed within each cohort). For the healthy cohort, the population-level and cohort-specific thresholds produce identical class splits (prevalence = 18.0% for physical fatigue), because the healthy cohort’s fatigue distribution closely matches the population mean. This confirms that the healthy-cohort result may not be confounded by between-cohort baseline differences and reflects within-person sleep-fatigue associations. For disease cohorts, cohort-specific thresholds substantially reduced positive-class prevalence. In the IMID cohort, prevalence of high physical fatigue dropped from 39.5% (population threshold) to 15.4% (cohort-specific), while in the NDD cohort, it dropped from 12.8% to 2.6% for DREEM-derived physical fatigue corresponding to a single positive sample in 39 recording nights. Given the extreme class imbalance and constrained sample sizes observed in certain cohorts, the validity of binary classification as a predictive framework is substantially undermined, as models trained under such conditions are prone to bias toward the majority class. Moreover, fatigue perception and baseline levels may differ substantially across individuals and clinical cohorts, such as those with IMID and NDD. Applying a single global threshold across heterogeneous cohorts may therefore obscure meaningful within-person fluctuations and cohort-specific fatigue patterns. We plan to address this limitation in our ongoing Clinical Observation Study (COS), which includes a substantially larger patient population. In additional, alternative thresholds (e.g., median split, clinical cutoffs) will be explored in future study. This will enable the evaluation of within-person and cohort-specific fatigue thresholds across different clinical groups (e.g., IMID and NDD) and allow a more robust assessment of class imbalance handling strategies. Another limitation in our current study is the class imbalance based on the global threshold method. We also evaluated resampling approaches, including the Synthetic Minority Oversampling Technique (SMOTE); however, these methods did not improve model performance.

Furthermore, several potentially important confounding variables, including pain, medication timing, disease activity, and mood, may influence fatigue but could not be systematically incorporated into the predictive models due to the limited sample size. In addition, prior-day measures such as physical activity, gait, daytime physiological indicators, and previous fatigue or sleepiness levels were excluded from the analysis. These factors may contribute substantially to next-day symptom variability and should be incorporated in future models.

Finally, the machine-learning models were developed and evaluated using data from the same study without a fully independent external test set. Although internal resampling methods such as cross-validation provide estimates of predictive performance, they may lead to optimistic bias due to overfitting or dataset-specific characteristics, including cohort composition, device usage patterns, and site procedures. In addition, several sleep-derived features are inherently correlated (e.g., sleep duration, efficiency, and stage proportions). Such feature collinearity may influence model stability and interpretation, particularly for models sensitive to multicollinearity. Although tree-based models are generally more robust to correlated predictors, future work could explore feature selection or dimensionality reduction approaches to better characterise independent contributions of sleep features. Consequently, the reported predictive performance should be considered preliminary and may not generalise to other populations, settings, or devices.

Future research will address these limitations using data from the ongoing COS, which includes a substantially larger and more diverse patient population. The expanded dataset will improve statistical power and enable the development of multimodal predictive models that integrate sleep physiology with daily activity measures (e.g., steps, activity intensity, sedentary time, walking bouts, and gait features) and symptom-related covariates. Incorporating circadian-related metrics, such as sleep timing, sleep regularity, and proxies for social jet, lag may also improve prediction of next-day fatigue and sleepiness.

In addition, future modelling approaches will focus on capturing within-person changes in symptoms, for example by modelling day-to-day differences in fatigue and sleepiness rather than absolute PRO levels. Such approaches may better align subjective reporting with physiological measures and reduce inter-subject response preference effects. Larger cohort sizes will also allow evaluation of cohort-specific or personalised fatigue thresholds and more robust strategies for handling class imbalance.

Future analyses will include a held-out independent test set to enable proper external validation, evaluation of model calibration, and assessment of subgroup performance and clinical utility. Moreover, time-series modelling approaches, including deep learning methods, will be explored to capture temporal relationships beyond the sleep period and examine the bidirectional dynamics between pre-sleep symptoms, sleep quality, and next-day fatigue and sleepiness. These efforts will help clarify the potential causal links between sleep physiology and fatigue and support the development of robust digital endpoints suitable for clinical research.

## 6 Conclusions

This preliminary feasibility study provides the first combined evaluation of wearable sleep tracker validity and next-day fatigue prediction in individuals with neurodegenerative and immune-mediated inflammatory diseases alongside healthy adults. Three consumer- and research-grade sleep trackers demonstrated acceptable agreement with polysomnography, supporting their suitability for long-term home-based sleep monitoring. Sleep-derived features showed preliminary, exploratory discriminative capacity for next-day physical fatigue in healthy adults, with cardiorespiratory features during sleep as the most informative candidate predictors.

Predictive performance differed substantially across cohorts, with models performing better in healthy adults than in chronic disease populations. A critical constraint on interpreting disease-cohort results is the use of a global (population-level) fatigue threshold, which conflates between-cohort baseline differences with within-person symptom variation; this is a primary explanation for limited performance in NDD and IMID groups, not simply device validity or heterogeneous fatigue mechanisms. Additionally, disease cohort subgroups, particularly NDD (n=29), are substantially underpowered, rendering subgroup AUC estimates and SHAP-derived feature rankings exploratory and hypothesis-generating only. These findings do not constitute evidence for disease-specific digital biomarkers of fatigue.

Physical and mental fatigue exhibited distinct predictive profiles, consistent with their treatment as partially separate constructs. While predictive capacity for fatigue-related PROs remains limited overall and is further constrained by outcome definition methodology in disease cohorts, the complementary value of objective wearable sleep tracking as a continuous monitoring tool remains scientifically justified. Together, these findings provide a methodological and exploratory empirical foundation for the rigorous, adequately powered evaluation planned in the larger Clinical Observation Study, which will address current limitations through within-person outcome modeling, cohort-specific thresholds, and substantially larger disease-cohort samples before any claims about disease-specific digital biomarkers can be substantiated.

## Data Availability

The original contributions presented in the study are included in the article/Supplementary Material, further inquiries can be directed to the corresponding author/s.

## Footnotes

^1^https://idea-fast.eu/ (accessed June 19, 2024).

^2^https://www.elive.fi/?lang=en (accessed June 19, 2024).

^3^https://www.zkboyue.com/en/index.html (accessed June 19, 2024).

^4^https://beacon.bio/dreem-headband/ (accessed June 19, 2024).

## IDEA-FAST Project Consortium

This communication reflects the view of the authors and/or IDEA-FAST Consortium and neither IMI nor the European Union and EFPIA are liable for any use that may be made of the information contained herein.
