# Supplementary material for: Exploring the feasibility of modeling next-day fatigue and sleepiness using digital sleep tracker data in neurodegenerative and immune-mediated inflammatory diseases
Source: Front Digit Health. 2026 Jun 17;8:1752629. doi: 10.3389/fdgth.2026.1752629 (PMC13318974; doi:10.3389/fdgth.2026.1752629)
Supplement: Supplementary file 1 [file Presentation1.pdf]

supplementary Materials

Bing Zhai[1,9†], Luan Chen[2,10†], Xujun Ma[2†], Clémence Pinaud[3], Meenakshi Chatterjee[4], Juha M. Kortelainen[5], Rana Zia Ur Rehman[14], Teemu Ahmaniemi[5], Stefan Avey[6], Yu Guan[16], Victoria Macrae[1], Chloe Hinchliffe[1], Silvia Del Din[1, 15], Nikolay V Manyakov[7], Robert Göder[16], Robbin Romijnders[8], Walter Maetzler[8], Ralf Reilmann[11], Svenja Aufenberg[11], Robin Schubert[11], C. Janneke van der Woude[12], Daqing Zhang[2], Wan-Fai Ng[1*], IDEA-FAST project consortium

[1]Translational and Clinical Research Institute, Faculty of Medical Sciences, Newcastle University, Newcastle upon Tyne, United Kingdom.
[2]SAMOVAR, Télécom SudParis, Institut Polytechnique de Paris, Palaiseau, France.
[3]Let it Care, France.
[4]Johnson & Johnson, Cambridge, MA, USA.
[5]VTT Technical Research Centre of Finland Ltd., Espoo, Finland.
[6]Johnson & Johnson, Spring House, PA, , USA.
[7]Johnson & Johnson, Beerse, Belgium.
[8]Department of Neurology, University Hospital Schleswig-Holstein, Kiel University, Kiel, Germany.
[9]Computer and Information Sciences, Northumbria University, Newcastle upon Tyne, United Kingdom.
[10]ETIS UMR8051, CY Cergy Paris Université, ENSEA, CNRS, Cergy, France.
[11]George Huntington Institute, University of Münster, Münster, Germany.
[12]Erasmus MC, Rotterdam, Netherlands.
[13]The Newcastle upon Tyne Hospitals NHS Foundation Trust, Newcastle upon Tyne, United Kingdom.
[14]Johnson & Johnson, Buckinghamshire, United Kingdom.
[15]National Institute for Health and Care Research (NIHR), Newcastle Biomedical Research Centre (BRC), Newcastle University, Newcastle upon Tyne, United Kingdom.

[16]Department of Computer Science, University of Warwick, Coventry, United Kingdom.
[17]Department of Psychiatry, University Hospital Schleswig-Holstein, Kiel University, Kiel, Germany.

*Corresponding author(s). E-mail(s): wanfai.ng@nhs.net;
Contributing authors: bing.zhai@Northumbria.ac.uk; luan.chen@ensea.fr; xujun.ma@telecom-sudparis.eu; clemence.pinaud@letitcare.com; mchatte4@ITS.JNJ.com; juha.m.kortelainen@vtt.fi; rrehman5@ITS.JNJ.com; Teemu.Ahmaniemi@vtt.fi; SAVEY@ITS.JNJ.com; Yu.Guan@warwick.ac.uk; victoria.macrae@newcastle.ac.uk; chloe.hinchliffe@newcastle.ac.uk; silvia.del-din@newcastle.ac.uk; nmanyak1@its.jnj.com; robert.goeder@uksh.de; r.romijnders@neurologie.uni-kiel.de; w.maetzler@neurologie.uni-kiel.de; ralf.reilmann@ghi-muenster.de; svenja.aufenberg@ghi-muenster.de; robin.schubert@ghi-muenster.de; c.vanderwoude@erasmusmc.nl; daqing.zhang@telecom-sudparis.eu; ;
†These authors contributed equally to this work.

1 Description of Research- and Consumer-Grade Sleep Trackers Used in the Study

BedSensor is a force-sensitive piezo-electric film that is placed under the mattress during sleep. The sensor stems from research from VTT Technical Research Centre in Finland and is commercialized through a partner organisation, eLive. The sensor detects heart rate, breathing rate, breathing disturbances and movements of the person lying on the mattress any time during the data. Prior research has validated BedSensor against gold standard references concerning breathing and sleep stages in a healthy population [1, 2]. BedSensor is CE-marked under the VitalTracker device name. The main resulting file from BedSensor is a hypnogram with sleep stages as ”deep sleep”, ”light sleep”, ”rapid eye movement (REM) sleep”, and ”wake” assesses for each epoch of length 60 seconds.

The ZKONE (YOLI Sleep Monitor) uses an Ultra-Wide Band signal (radar) to monitor and record respiratory rate, heart rate, sleep staging and body movement passively and unobtrusively. It can sense body movement up to three meters from the device and can detect a range of sleep conditions, such as sleep apnoea and staging. The device should be placed next to a bed at waist height. ZKONE is designed to only analyze data recorded from 4 p.m. to the next day’s 10 a.m., thus only night sleep. Prior work has validated ZKONE against gold standard for respiration rate in healthy population [3], while recent work has used ZKONE in clinical contexts to

screen COVID patients [4]. The main resulting file from ZKONE is a hypnogram with sleep stages as "deep sleep", "light sleep", "rapid eye movement (REM) sleep", and "wake" assessed in epochs of length 60 seconds.

DREEM 2 is a wireless headband worn during sleep that records physiological data in real time. Five types of physiological signals are recorded via three types of sensors embedded in the device: brain cortical activity via five EEG dry electrodes; movements, position, and breathing frequency via a 3-D accelerometer located over the head; heart rate via a red-infrared pulse oximeter located in the frontal band. The DREEM 2 has been clinically validated in prior work [5] and is often used to monitor individuals who experience sleep disturbances [6]. DREEM 2 is CE marked. The main resulting file from DREEM 2 is a hypnogram with sleep stages as N1, N2, N3, REM and wake assessed in epochs of length 30 seconds.

2 Patient reported outcomes collected with the Stress Monitor Application

Table 1: Patient reported outcomes (PROs) collected with the Stress Monitor Application.

**PRO**	**Type**	**Questionnaire time**			
		**Morning (9–12)**	**Early afternoon (13–16)**	**Late afternoon (17–20)**	**Evening (21–23:30)**
Physical fatigue	Likert item	X	X	X	X
Mental fatigue	Likert item	X	X	X	X
Sleepiness, current feeling	Drop-down menu		X	X	X
I went to bed at	Clock	X			
I woke up at	Clock	X			
How was your sleep?	Likert item	X			
Time to fall asleep	Drop-down menu	X			
Time awake during night	Drop-down menu	X			

3 Sleep characteristics obtained from PROs

Table 2: Description of PROs features

**Featue code**	**Feature description**
Physical fatigue	The highest self-reported physical fatigue level recorded throughout the day, following the use of a sleep tracker in the previous night. The likert scale ranges from 0 to 6, representing levels from low to high.
Mental fatigue	The highest self-reported mental fatigue level recorded throughout the day, following the use of a sleep tracker in the previous night. The likert scale ranges from 0 to 6, representing levels from low to high.
Sleepiness	The highest self-reported sleepiness level recorded throughout the day, following the use of a sleep tracker in the previous night. The likert scale ranges from 0 to 9, representing levels from extremely alert to extremely sleepy .
Sleep duration	The time difference between the participant's self-reported sleep onset ("Time to fall asleep...") and wake-up time ("I woke up at...").
Sleep quality	A numerical rating provided by the participant in response to the question "How was your sleep ?". The Likert scale ranges from 0 to 6, representing levels from bad to excellent.
Fall sleep time	The numerical value provided by the participant in response to the question "Time to fall asleep...".
Wake duration	Participant's estimated total wake time between sleep onset and sleep offset, selecting from one of six predefined categories: "less than 15 minutes," "15 to 30 minutes," "30 minutes to 1 hour," "1 to 2 hours," "2 to 3 hours," and "more than 3 hours."

4 Feature interpretation of Sleep trackers for association analysis and machine learning modeling

Table 3: The sleep-related metrics derived from ZKONE.

Feature code	Featue Name	Measurement/Calculation Method
Z_HR_q05	Sleep HR BPM q05	95% confidence intervals of the heart rate mean (lower bound).
Z_HR_q95	Sleep HR BPM q95	95% confidence intervals of the heart rate mean (upper bound).
Z_HR_mean	Sleep HR Mean	Sample mean of heart rate during sleep.
Z_HR_std	Sleep HR Std	Sample standard deviation of heart rate.
Z_RR_q05	Sleep RR BPM q05	95% confidence intervals of the respiration rate mean (lower bound).
Z_RR_q95	Sleep RR BPM q95	95% confidence intervals of the respiration rate mean (upper bound).
Z_RR_mean	Sleep RR Mean	Sample mean of respiration during sleep.
Z_RR_std	Sleep RR Std	Sample standard deviation of respiration rate.
Z_TST	Total Sleep Time (sec)	Duration from sleep onset till sleep awakening.
Z_WASO_duration	Wake After Sleep Onset (WASO) (sec)	The total awake time after sleep onset until sleep awakening.
Z_WASO_ratio	WASO Ratio	Wake After Sleep Onset / Total Sleep Time
Z_WASO_times	WASO Times	The number of WASO during sleep.
Z_Fall_asleep_time	Fall Asleep Time (sec)	Duration from light-off time to sleep onset time.
Z_Last_wake_duration	Last Wake Duration	Duration of the last wake period
Z_AHI	Apnea Hypopnea Index	AHI is calculated using a proprietary algorithm
Z_Turnover	Turnover	The number of body turnovers during the sleep period.
Z_Sleep_efficiency	Sleep efficiency	Duration of (REM+Light+Deep) sleep/Total Sleep Time
Z_Sleep_score	Sleep Score	Derived from a proprietary algorithm.
Z_LIGHT_ratio	Light Sleep Ratio	Light Sleep Duration/Total Sleep Time
Z_LIGHT_duration	Light Sleep Duration (sec)	Total duration spent in N1 and N2 sleep stages.
Z_DEEP_ratio	Deep Sleep Ratio	Deep Sleep Duration/Total Sleep Time
Z_DEEP_duration	Deep Sleep Duration (sec)	Total duration spent in N3 sleep stage.
Z_REM_duration	REM Duration (sec)	The total amount of time spent in the rapid eye movement sleep throughout the sleep period.
Z_REM_ratio	REM Ratio	REM Duration / Total Sleep Time

Table 4: The sleep-related metrics derived from BedSensor.

**Feature Code**	**Featue Name**	**Measurement/Calculation Method**
B_HR_q05	Sleep HR BPM q05	5% quantile of heart rate during sleep.
B_HR_q90	Sleep HR BPM q90	90% quantile of heart rate during sleep.
B_HR_q80	Sleep HR BPM q80	80% quantile of heart rate during sleep.
B_HR_q95	Sleep HR BPM q95	95% quantile of heart rate during sleep.
B_HR_median	Sleep HR Median	The median of heart rate during sleep.
B_HR_mean	Sleep HR Mean	The mean of heart rate during sleep.
B_HR_std	Sleep HR Std	The standard deviation of heart rate during sleep.
B_RR_q05	Sleep RR BPM q05	5% quantile of respiration during sleep.
B_RR_q90	Sleep RR BPM q90	90% quantile of respiration during sleep.
B_RR_q80	Sleep RR BPM q80	80% quantile of respiration during sleep.
B_RR_q95	Sleep RR BPM q95	95% quantile of respiration during sleep.
B_RR_median	Sleep RR Median	The median of respiration during sleep.
B_RR_mean	Sleep RR Mean	The mean of respiration during sleep.
B_RR_std	Sleep RR Std	The standard deviation of respiration during sleep
B_TST	Total Sleep Time (sec)	Duration from sleep onset till sleep awakening.
B_WASO_duration	Wake After Sleep Onset (WASO) (sec)	The total awake time after sleep onset until sleep awakening.
B_WASO_ratio	WASO Ratio	Wake After Sleep Onset/Total Sleep Time
B_WAKE_times	WASO Times	The number of WASO during sleep.
B_WAKE_before_s	Sleep Latency (sec)	Duration from light-off time to sleep onset time.
B_WAKE_longest_s	Longest wake during sleep (sec)	The longest weak period during total sleep time.
B_Sleep_efficiency	Sleep efficiency	Duration of (REM+Light+Deep) sleep/Total Sleep Time
B_Sleep_quality	Sleep Quality	The value is calculated using a function of sleep duration and sleep hypnogram.
B_LIGHT_ratio	Light Sleep Ratio	Light Sleep Duration / Total Sleep Time
B_LIGHT_duration	Light Sleep Duration (sec)	Total duration spent in N1 and N2 sleep stages.
B_DEEP_ratio	Deep Sleep Ratio	Deep Sleep Duration / Total Sleep Time
B_DEEP_duration	Deep Sleep Duration (sec)	Total duration spent in N3 sleep stage.
B_REM_duration	REM Duration	The total amount of time spent in the rapid eye movement sleep throughout the sleep period
B_REM_ratio	REM Ratio	REM Duration / Total Sleep Time

Table 5: The sleep-related metrics derived from DREEM 2.

Feature code	Feature Name	Measurement/Calculation Method
D_HR_mean	Mean Heart Rate per Sleep	The mean of heart rate during sleep.
D_RR_mean	Mean Respiration Rate per Sleep	The mean of respiration rate during sleep.
D_TST	Total Sleep Time (sec)	Duration from sleep onset till sleep awakening.
D_WASO_duration	Wake Duration After Sleep Onset (WASO) (sec)	The total awake time after sleep onset until sleep awakening.
D_Waso_ratio	WASO Ratio	Wake After Sleep Onset / Total Sleep Time
D_Fall_asleep_time	Sleep Latency	Duration from light-off time to sleep onset time.
D_Turnover	Turnover	The number of body turnovers during the sleep period.
D_Sleep_efficiency	Sleep Efficiency	Duration of (REM+N1+N2+N3) sleep/Total Sleep Time
D_NREM_ratio	NREM Ratio	NREM durantion / Total Sleep Time
D_NREM_duration	NREM Duration	Total duration spent in the NREM sleep stages.
D_N1_duration	N1 Duration	The total amount of time spent in the N1 throughout the sleep period.
D_N1_ratio	N1 ratio	N1 Duration / Total Sleep Time
D_N2_duration	N2 Duration	The total amount of time spent in the N2 throughout the sleep period.
D_N2_latency	N2 Latency	Time required to transition from wakefulness to the N2 sleep stage.
D_N2_ratio	N2 ratio	N2 Duration / Total Sleep Time
D_N3_duration	N3 Duration	The total amount of time spent in the N3 throughout the sleep period.
D_N3_latency	N3 Latency	Time required to transition from wakefulness to the N3 sleep stage.
D_N3_ratio	N3 ratio	N3 Duration / Total Sleep Time
D_REM_duration	REM Duration	The total amount of time spent in the rapid eye movement sleep throughout the sleep period
D_REM_ratio	REM Ratio	REM Duration / Total Sleep Time
D_REM_latency	REM Latency	Time required to transition from wakefulness to the REM sleep stage.

5 Hyperparameters of machine learning model used during the tuning

Table 6: The hyperparameter configuration for each model

Classifier	Hyperparameter Tuning Setup
Logistic Regression	Regularisation: {L1, L2, elastic net, no regularisation}
	Optimiser: {Coordinate descent (LIBLINEAR), stochastic average gradient decent (SAGA)}
Random Forest	Number of trees: {50, 100, 200, 300, 400},
	Maximum of features for the best split: {Square root of the number of features (SQRT)}
	Minimum samples to split: {2}
	Criterion: {Gini}
	Maximum Depth: {None}
ReLU	Optimiser: {Stochastic gradient descent (SGD), adaptive Moment Estimation optimizer(Adam)}
	Learning Rate: {Adaptive}
	Hidden layer size: {8, 16, 32}
	Activation function for the hidden layer: {ReLU}
	Number of layers: {2}
	L2 regularization alpha: {0.1, 0.01}

6 Metrics for assesment of modeling performance

Table 7: Assessing Classification Model Performance Through Metrics.

**Measure**	**Formula**	**Function, Focus**
Precision (Positive Predictive Value)	$\frac{TP}{TP+FP}$	Agreement between the data labels and positive labels given by the algorithm.
Recall (Sensitivity)	$\frac{TP}{TP+FN}$	Effectiveness of the algorithm to identify positive labels.
Specificity	$\frac{TN}{TN+FP}$	Effectiveness of the algorithm to identify negative labels.
F1 Score	$\frac{2 \cdot \text{Precision} \cdot \text{Recall}}{\text{Precision}+\text{Recall}}$	In multi-sleep stage classification, each stage is treated as an individual binary classification issue (specific stage versus all other stages), and the F1 score for each stage is computed separately.
Macro F1 Score	$\text{Macro-F1} = \frac{1}{N}\sum_{i=1}^{N} F1_i$	Calculate the F1 score independently for each class and then take the average.
Optimal Cut Point	Youden's index $J(c) = TPR(c) + FPR(c)$ [7]	The cut-point c optimizes the digital measure's differentiating ability when equal weight is given to sensitivity and specificity. Optimal Cut Point is : $c^* = argmax_c J(c)$

References

[1] Kortelainen, J.M., Van Gils, M., Pärkkä, J.: Multichannel Bed Pressure Sensor for Sleep Monitoring. In: 2012 Computing in Cardiology, pp. 313–316 (2012). IEEE

[2] Mendez, M.O., Palacios-Hernandez, E.R., Alba, A., Kortelainen, J.M., Tenhunen, M.L., Bianchi, A.M.: Detection of the sleep stages throughout non-obtrusive measures of inter-beat fluctuations and motion: night and day sleep of female shift workers. Fluctuation and Noise Letters **16**(04), 1750033 (2017)

[3] Raheel, M.S., Coyte, J., Tubbal, F., Raad, R., Ogunbona, P., Patterson, C., Perlman, D.: Breathing and Heartrate Monitoring System using IR-UWB Radar. In: 2019 13th International Conference on Signal Processing and Communication Systems (ICSPCS), pp. 1–5 (2019). IEEE

[4] Dong, C., Qiao, Y., Shang, C., Liao, X., Yuan, X., Cheng, Q., Li, Y., Zhang, J., Wang, Y., Chen, Y., *et al.*: Non-contact Screening System based for COVID-19 on XGBoost and Logistic Regression. Computers in Biology and Medicine **141**, 105003 (2022)

[5] Arnal, P.J., *et al.*: The Dreem Headband Compared to Polysomnography for Electroencephalographic Signal Acquisition and Sleep Staging. Sleep **43**(11), 097 (2020) https://doi.org/10.1093/sleep/zsaa097

[6] Waeber, A., Arnal, P.J., Lecciso, G., Albir, D., Mignot, E., Heinzer, R.: Acoustic Stimulation Time-locked to the Beginning of Sleep Apnea Events Reduces Oxygen Desaturations: A Pilot-study. Sleep Medicine **78**, 38–42 (2021)

[7] Youden, W.J.: Index for rating diagnostic tests. Cancer **3**(1), 32–35 (1950)
